# Efficient quantum gates and algorithms in an engineered optical lattice

**DOI:** 10.1038/s41598-021-94929-4

**Published:** 2021-07-28

**Authors:** A. H. Homid, M. Abdel-Aty, M. Qasymeh, H. Eleuch

**Affiliations:** 1 Mathematics Department, Faculty of Science, Al-Azhar University, Assiut, 71524 Egypt; 2 Mathematics Department, Faculty of Science, Sohag University, Sohag, 82524 Egypt; 3 Electrical and Computer Engineering Department, Abu Dhabi University, Abu Dhabi, UAE; 4 Department of Applied Physics and Astronomy, University of Sharjah, Sharjah, UAE; 5 Institute for Quantum Science and Engineering, Texas A&M University, College Station, TX 77843 USA

**Keywords:** Quantum physics, Applied mathematics, Information technology

## Abstract

In this work, trapped ultracold atoms are proposed as a platform for efficient quantum gate circuits and algorithms. We also develop and evaluate quantum algorithms, including those for the Simon problem and the black-box string-finding problem. Our analytical model describes an open system with non-Hermitian Hamiltonian. It is shown that our proposed scheme offers better performance (in terms of the number of required gates and the processing time) for realizing the quantum gates and algorithms compared to previously reported approaches.

## Introduction

Ultracold atoms in optical lattices enjoy high degrees of controllability and long decoherence time. For example, the exotic interactions in many-body systems of matter can control the optical lattice and probe high order quantum phenomena^1,2^. Furthermore, ultracold atoms in optical lattices can incorporate various types of interactions. These include lattice defects, electron–electron interactions, electron–phonon interactions, and spin-orbital coupling (i.e., SOC)^3^. Therefore, several reports have investigated the use of ultracold atoms in optical lattices to study strongly correlated quantum systems^4–8^.

Normally, ultracold atoms in optical lattices adhere superfluid state and provide lattice disorders. However, mott-insulator regime can be obeyed for small tunneling rates between adjacent atoms, and uniform lattice structure can be obtained^9,10^. Interestingly, such a structure provides promising platform for quantum gates and quantum algorithms^11,12^.

In Refs.^13–19^ quantum gates have been realized using optical lattices. Furthermore, in Refs.^20–22^ trapped atoms in optical lattices have been functioned to achieve quantum gates and algorithms. We consider in this work the case of trapped ultracold atoms that incorporate spin-orbital coupling and Zeeman splitting. Accordingly, we show that quantum gates and algorithms can be realized based on our scheme with better performance (in term of processing time) as compared with the previously reported approaches. For instance, we investigate the realization of controlled-not gate and Toffoli gate circuit using the proposed scheme. Also, Simon algorithm^23^ and black-box string finding algorithm are proposed and evaluated. It is shown that the number of required gates (and the processing time needed) to implement such gates and algorithms is significantly smaller than previously reported realizations.

The outline of the manuscript is as follows: In “The model”, we introduce model and the Hamiltonian. In “New circuits in engineered lattices”, we present the quantum gate circuits and discuss their performance. “Quantum algorithms” is dedicated for the quantum algorithm schemes. Finally, “Conclusion” includes the concluding remarks.

## The model

We consider a system of bosonic (or fermionic) ultracold atoms that are trapped in a square optical lattice and subjected to spin-orbital coupling (SOC) and Zeeman field (ZF) mechanisms^24–27^. We take in the consideration the following effects: (i) an anisotropic Dzyaloshinskii–Moriya interaction (DMI) in three dimensions; (ii) an engineered dissipation causing the decay of the dipole of only boson atoms and the decay of dipole–dipole interaction of the boson and fermion atoms. Analytically, the dissipation of the dipole–dipole interaction and 3D DMI have not been previously studied for interacting atoms inside a lattice.

The setup of the physical system and the related Hamiltonian are detailed in the Supplementary Information (Supplementary Section S1). Using the iso-spin operators, $2\vec {S}_{v}=\sum _{k,k^{'}}\hat{a}^{\dagger }_{vk}\hat{\sigma }_{kk^{'}}\hat{a}_{vk^{'}}$, with $\hat{\sigma }_{kk^{'}}$ indicates the elements of Pauli matrices for each corresponding spin operator and $\hat{a}_{vk}$ represents the atom lowering operator of spin state *k* at site *v*, the the non-Hermitian Hamiltonian can be expressed as follow:

1 $$\begin{aligned} \hat{H}= & {} \sum _{v,j=1}^{\wp }\vec {S}_{v}\cdot \mathbf{J} \cdot \vec {S}^{\tau }_{j}+\sum _{v,j=1}^{\wp }\vec {D}_{vj}\cdot (\vec {S}_{v}\times \vec {S}_{j})-\sum _{v=1}^{\wp }f_{z}\hat{S}^{z}_{v}-i\gamma _{1}\sum _{v=1}^{\wp }\hat{S}^{+}_{v}\hat{S}^{-}_{v}\nonumber \\&-i\gamma _{2}\sum _{v, j=1}^{\wp }(\hat{S}^{+}_{v}\hat{S}^{-}_{j}+\hat{S}^{-}_{v}\hat{S}^{+}_{j}) -4i\gamma _{2}\sum _{v\ne j}^{\wp }(\hat{S}^{+}_{v}\hat{S}^{-}_{j})(\hat{S}^{+}_{j}\hat{S}^{-}_{v})+\aleph \hat{I}, \end{aligned}$$

where $\wp$ is the number of sites, $\vec {S}_{v}=\{\hat{S}^{x}_{v},\hat{S}^{y}_{v},\hat{S}^{z}_{v}\}$ is the vector of spin operators, $\tau$ refers to the transpose, $\mathbf{J} =\text {diag}\{J_{x},J_{y},J_{z}\}$ is the matrix of the exchange couplings, $\vec {D}_{vj}=(D_{x},D_{y},D_{z})$ indicates the coefficients vector of DMI, $f_{z}$ is the coefficient of Zeeman field, $\gamma _{1}$ is the dissipation parameter of the dipole for Boson atoms, $\gamma _{2}$ represents the dissipation parameter of the dipole-dipole interaction of the Boson and Fermion atoms, and $\aleph$ is a constant. Here, $\gamma _{1}<\gamma _{2}$ and $f_{z}\ne J_{z}$. More details can be found in Supplementary Section S1 of the Supplementary Information.

We note that the coupling rates (i.e., $J_{x}$, $J_{y}$ and $J_{z}$) in the Hamiltonian (1) can be controlled by adjusting the SOC, ZF and the tunneling parameters. For instance, in the absence of SOC and ZF mechanisms, the $J_{x},J_{y}$ are positive (negative) and $J_{z}$ is negative (positive) for the bosonic (fermionic) atoms. However, in the case of incorporating the SOC and the ZF mechanisms, all possible combinations of the coupling rate values can be found. In other words, in the absence of the SOC and ZF effects, the trapped atoms behave as ferromagnetic and antiferromagnetic mediums for bosonic and fermionic atoms, respectively. In contrast, for the case of having the SOC and ZF effects, one can design the behaviour of the ultracold trapped atoms to be either antiferromagnetic or ferromagnetic mediums for any type of the atoms. Interestingly, the SOC, ZF and tunneling parameters can be controlled by several operational parameters including the intensity of the laser Raman beams, the external magnetic field and by adjusting the Feshbach resonance (via tuning the ratio between the scattering lengths)^28^.

## New circuits in engineered lattices

In this section, we construct efficient new circuits for controlled not (CN) and controlled-controlled not (CCN) gates based on the proposed scheme of trapped ultracold atoms. As opposed to all previously reported CN and CCN gates^13–19^, our proposal is considering a full Heisenberg chain with Zeeman field and DMI effect. Realizing CN and CCN gate based on the Hamiltonian (1) require a series of quantum gates. Similarly, earlier reported approaches for two-atom gates implement several one-atom gates^13–19^. It is important to note here that the single-atom gates can not be neglected in our proposed schemes as the single-atom gate forms a basic block for the proposed circuit schemes. Thus, the single-atom gates must be taken into account along with the two-atom gates when the cost of our proposed gate is evaluated. On the other hand, the SOC effect can be cancelled in ultracold atoms by controlling the laser beams and having very weak coupling with the lattice atoms. The dissipation in this case is omitted. This scenario has been demonstrated in Refs.^7,8^. However, in this work, we consider a generic approach in which the laser beams are strongly coupled with the atoms and the dissipation is existing. Nevertheless, the considered laser beams are yet functional to be tuned to omit dissipation. Importantly, we will show that by including the SOC effect, the cost of the quantum gates can be optimized for smaller values than the case of omitted SOC effect.

### Proposed circuit of CN-gate

**Figure 1:**
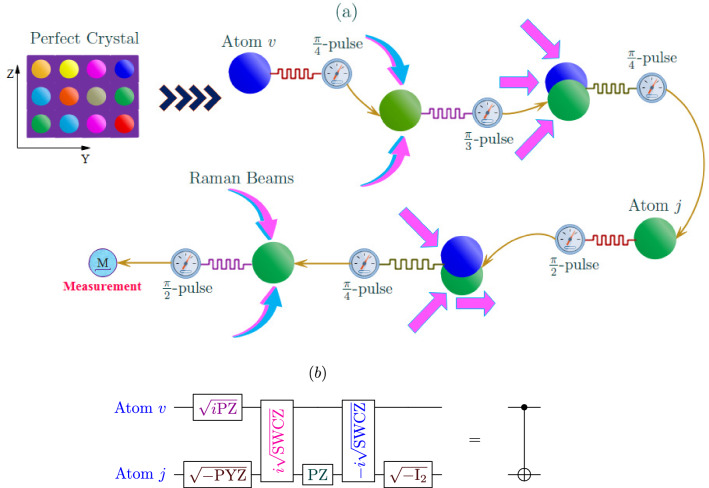
The proposed configuration of the CN gate circuit: (**a**) The operation layout of CN-gate setup. (**b**) The equivalent configuration of the CN circuit.

In the proposed scheme to construct the CN-gate circuit, two counter-propagating laser beams are applied to the ultracold atoms to create a non-defective square optical lattice (perfect crystal) with one atom occupying a site. The proposed circuit can be constructed using two ultracold atom pairs in the lattice (e.g., site *v* and *j*). The operation of the CN gate can be represented schematically in Fig. 1a.

As can be seen from the Supplementary Information (see Supplementary Section S2), atoms’ transitions in the proposed configuration can be functioned to form several quantum gates. These include the square root of $-\text {SWAP}$ and controlled-z gate ($\pm i\sqrt{\text {SWCZ}}$), the square root of the imaginary Pauli-Y and the square root of the imaginary Pauli-Z ($\sqrt{-\text {PYZ}}$), the square root of the minus identity operator ($\sqrt{-\text {I}_{2}}$), the square root of the imaginary Pauli-Z ($\sqrt{i\text {PZ}}$) and Pauli-Z ($\text {PZ}$). Furthermore, a combined transitions can be functioned to realize CN-gate, see Fig. 1b.

The performance of the obtained CN-gate circuit can be assessed by calculating the quantum cost (i.e, the number of required gates or the realization time) of the circuit and compared it with the cost of previously reported CN-gate realizations^13–18^. In Table 1, we present the quantum cost for our CN-gate along with previously reported approaches. It is clear that the number of the required gates (or realization time) for our circuit is less than those required by previous approaches. The considered work in Table 1 is for the case of including DMI effect. However, in absence of DMI effect^7,8^, the realization of all other approaches will require notably more gates. Nonetheless, our proposed circuit can be achieved in the absence of DMI effect with slightly more gates (see part 2D presented by Supplementary Information). Hereby, our results demonstrate an encouraging sign of providing a more efficient approach as compared to previously reported approaches that do not implement the DMI effect. This is a significant advantage of our proposal. We note that a large number of related recent studies can be found in the literature. However, all of these studies are based on the native circuits of Refs.^13–18^. Therefore, our work has been devoted to present two new circuits of CN and CCN gates that have an enhanced performance (in terms of the number of required gates and the processing time) compared to the circuits of Refs.^13–18^.

**Table 1:** CN-gate quantum cost (and realization time) for the current work and previous proposals.

CN circuits	Refs.	Required gates	Required times
$\sqrt{i\text {PZ}}$, $\sqrt{-\text {PYZ}}$, PZ, $\sqrt{-\text {I}_{2}}$, $\pm i\sqrt{\text {SWCZ}}$	This work	**6**	$\frac{2.592\pi \hbar }{\zeta }+\frac{\pi }{2\theta }$
2R$_{y}(\frac{\pi }{2})$, R$_{z}\left( \pm \frac{\pi }{2}\right)$, R$_{z}(\pi )$, $\text {Ph}(\vartheta _{R})$, R$_\mathbf{n _{1}}\left( \frac{\pi }{3}\right)$, R$_\mathbf{n _{2}}\left( \frac{\pi }{3}\right)$, $2\sqrt{i\text {SWAP}}$	^13^	**10**	$\frac{14\pi \hbar }{\zeta }+\frac{3\pi }{2\theta }+\tau _{1}$
R$_{x}\left( \pm \frac{\pi }{2}\right)$, 2R$_{z}\left( \pm \frac{\pi }{2}\right)$, R$_{z}(\pi )$, $2\sqrt{\text {SWAP}}$	^14,17^	**15**	$\frac{81\pi \hbar }{4\zeta }+\frac{9\pi }{2\theta }$
$2\mathcal {H}$, CZ	^15,16^	**7**	$\frac{17\pi \hbar }{4\zeta }+\frac{2\pi }{\theta }$
2R$_{x}\left( \frac{\pi }{2}\right)$, 2R$_{z}\left( \frac{\pi }{2}\right)$, R$_{z}\left( -\frac{\pi }{2}\right)$, $\text {Ph}(\vartheta _{R})$, $2i\text {SWAP}$	^17^	**8**	$\frac{29\pi \hbar }{2\zeta }+\frac{\pi }{\theta }$
$2\mathcal {H}$, 2R$_{z}\left( \frac{\pi }{2}\right)$,$\text {SWAP}$, $i\text {SWAP}$	^18^	**11**	$\frac{45\pi \hbar }{4\zeta }+\frac{4\pi }{\theta }$

### Proposed circuit of CCN-gate

**Figure 2:**
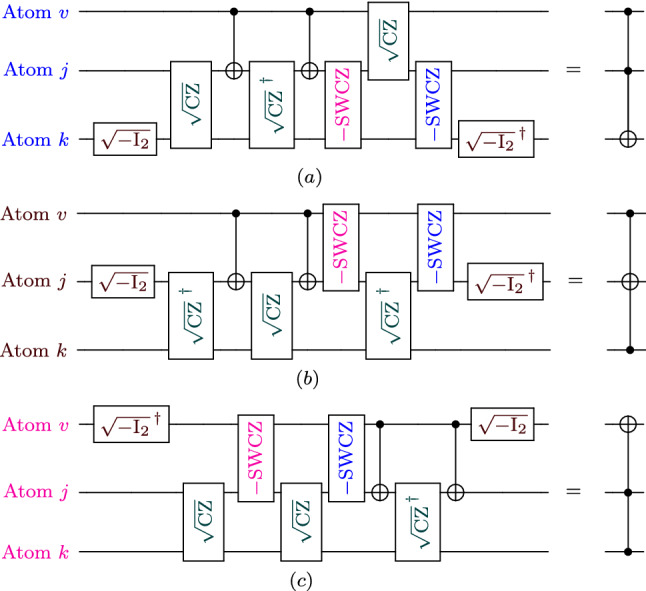
The proposed configuration of the CCN gate. Here, the cost for each of $\sqrt{\text {CZ}}$ and $\sqrt{\text {CZ}}^{\dagger }$ is three gates.

The circuit of CCN-gate can be realized by utilizing three atoms (i.e., *v*, *j* and *k*) inside the lattice. Such interaction can take place by incorporating two pair interactions: atoms *v* and *j* and atoms *j* and *k*. It then follows that the CCN-gate is similar to CN-gate but with extra procedures, such as including the square root of controlled-z ($\sqrt{\text {CZ}}$) gate and its inverse. The corresponding configurations of the CCN-gate is shown in Fig. 2.

The main advantage of our CCN-gate configuration is requiring less number of gates (and thus processing time) as compared to previously reported circuits^15,16^. For the sake of fair comparison between our CCN-gate and previous work, let us consider the CN-gate as one gate. Consequently, one can see that our proposed scheme requires 15 gates to construct the CCN-gate circuit for adjacent interacting atoms, while the required gates is even less than 15 for nonadjacent interacting atoms. However, for the reported work in Refs.^15,16^ with DMI effect, 18 and 17 gates are required for nonadjacent interacting atoms, and 34 and 25 for adjacent interacting atoms, respectively. Moreover, in absence of DMI effect, the number of required gates is much larger for the approaches in Refs.^15,16^, that for example require five and four gates for two-atom circuits, such as CN and SWAP gates, respectively.

### Challenges and obstacles

In this subsection, we address the non-ideal effects that would arise in the proposed scheme. One of these effects is the dissipation which causes an instability of the spin currents of the lattice atoms. On the other hand, the instability of the Mott-insulator, caused by the inaccuracy of the laser beams application to the atoms, leads to superfluid regions and local fluctuations due to atom-hole defects^29^. These unwanted dynamics can be suppressed by tuning the tunneling and temperature via adjusting the laser beams and utilizing proper experimental parameters^29^. The dissipation can be controlled by adjusting the Raman laser beams that are applied to the lattice.

The impact of dissipation on the gates’ performance can be described by the concept of fidelity. Interestingly, the fidelity in the proposed system and thus the robustness for each circuit to errors can be externally controlled. Using fidelity, one can compare the performance of the gates with and without dissipation. The gates fidelity can be defined by^30^:

2 $$\begin{aligned} \mathcal {F}_{\text {av}}(t,\gamma ,\gamma _{1},\gamma _{2}) = \frac{2^{\wp } +|\text {Tr}\{\mathfrak {U}^{-1}_{1}\mathfrak {U}_{2}(t,\gamma ,\gamma _{1},\gamma _{2})\}|^{2}}{2^{\wp }(2^{\wp }+1)}, \end{aligned}$$

where $\mathfrak {U}^{-1}_{1}$ is the inverse of the circuit function without dissipation, $\mathfrak {U}_{2}$ is the circuit function with dissipation. The CCN gate is controlled by the three interaction times. Our calculations show that the dissipation is mainly attributed to the dipole and dipole–dipole interactions.

**Figure 3:**
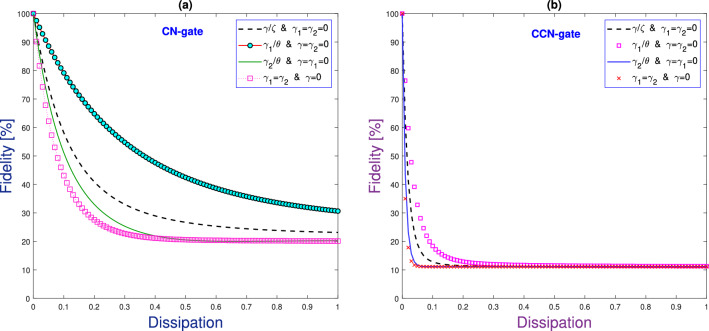
Gates fidelity against dissipation rates for CN and CNN gates.

**Figure 4:**
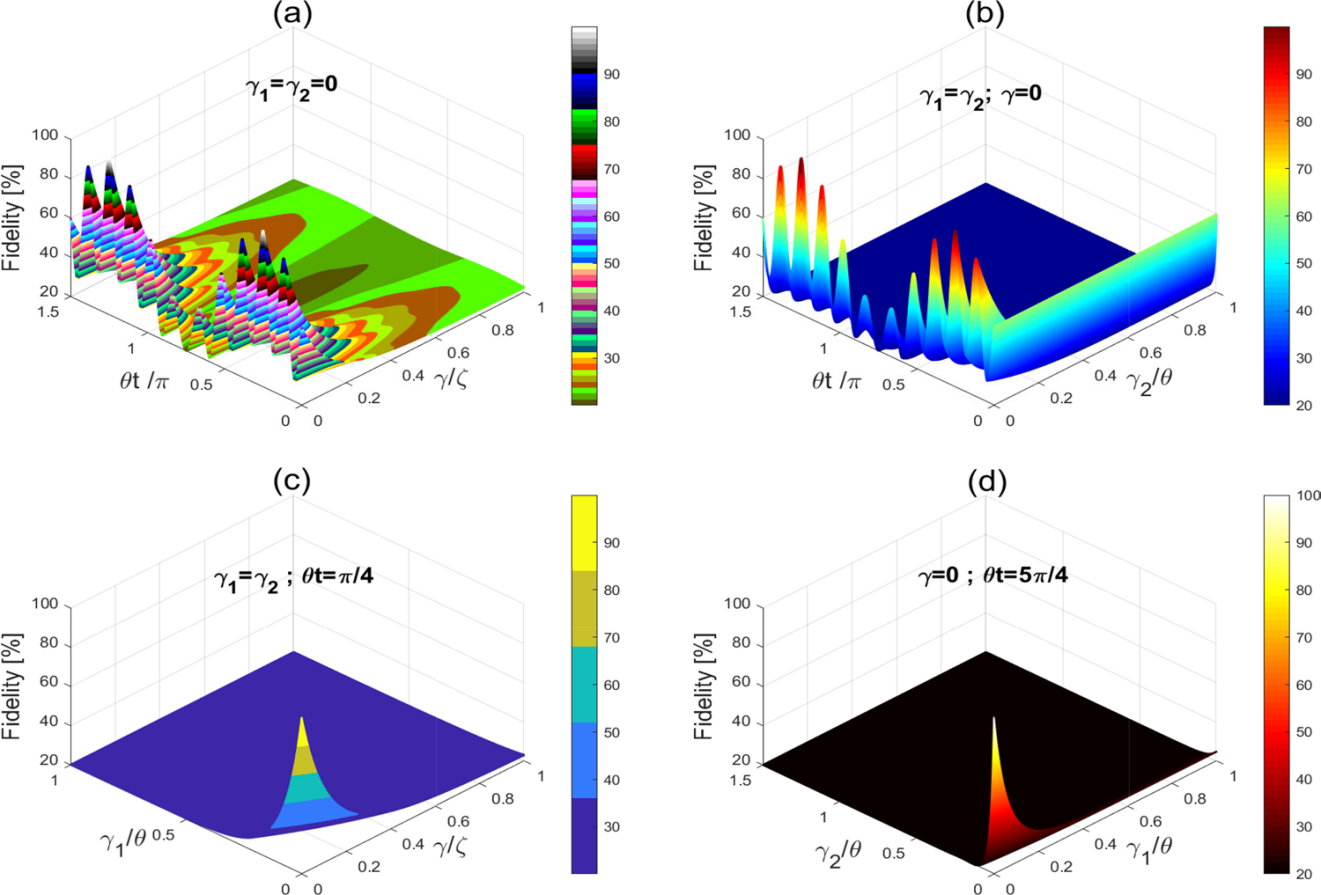
The CN gate fidelity versus the interaction time and dissipation rates. (**a**) Non-interacting atoms versus dissipation and interaction time. (**b**) Interacting atoms versus dissipation and interaction time. (**c**) Interacting and non-interacting atoms versus dissipation at $t=\frac{\pi }{4\theta }$. (**d**) Interacting and non-interacting atoms versus dissipation at $t=\frac{5\pi }{4\theta }$.

**Figure 5:**
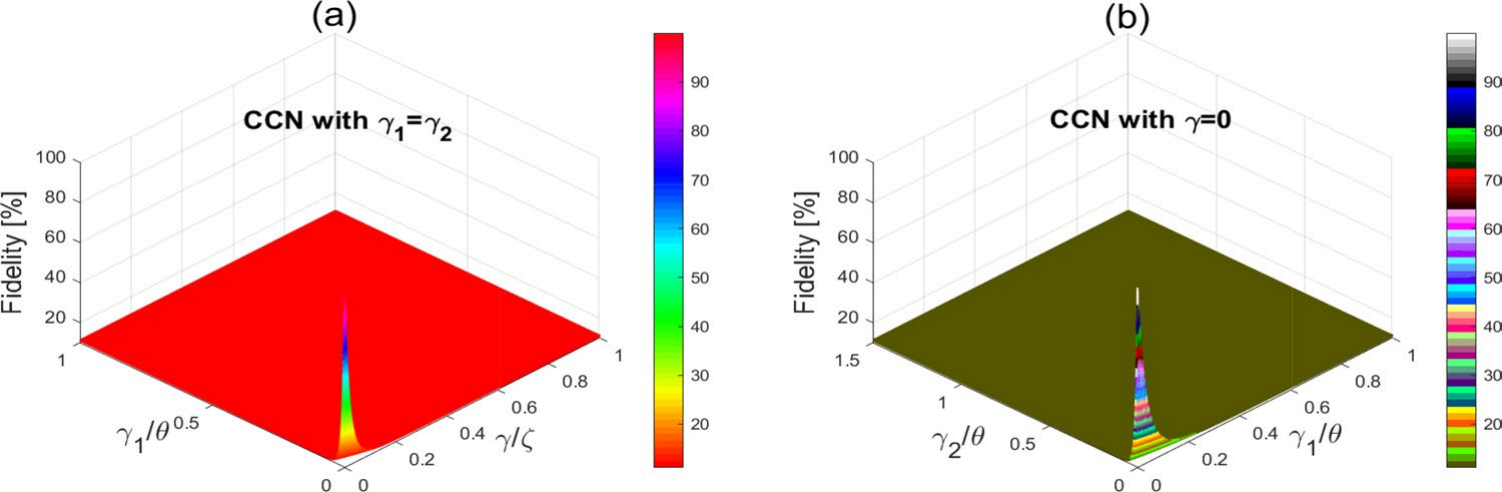
The CCN gate fidelity against dissipation.

Figure 3 displays the fidelity of the CN and CCN gates as a function of different dissipation rates. From this Figure, we note that the fidelity of the CN and CCN circuits can be at its maximum of 100% in absence of dissipation. In contrast, one can note that the fidelity is slightly decreased yet with acceptable levels (i.e., 75% $\sim$ 90%) in the presence of weak dissipation values (around 0.1). For larger dissipation rates, grater than 0.6 for CN and 0.2 for CCN gates, we observe that the fidelity is greatly decreased and the prediction of the success rates is not exceeding 31% and 12% for the CN and the CCN gates, respectively. Here, it is clearly seen that the fidelity of the CCN-gate is more sensitive to dissipation as compared to the CN-gate. Also, in Fig. 4a,b, we evaluate the fidelity of the CN gate versus the interaction time and dissipation. We note that the fidelity of the CN can be maintained at its maximum of 100% in the absence of dissipation for the interaction times $t=\frac{\pi (4j_{1}+1)}{4\theta }, j_{1}=0,1,2,...$. Interestingly, even in the presence of dissipation, and for $\frac{\gamma }{\zeta }<0.02$ and $\frac{\gamma _{2}}{\theta }<0.02$, the CN fidelity is slightly decreasing and can be maintained above 80%, given that the interaction times are properly chosen according to the above criterion. While for greater dissipation, such as $\frac{\gamma }{\zeta }>0.2$ and $\frac{\gamma _{2}}{\theta }>0.2$, the fidelity is significantly decreasing reaching levels below 21%. For instance, as can be seen from Fig. 4c,d, even at the interaction times $t=\frac{\pi (1)}{4\theta }$ and $\frac{\pi (5)}{4\theta }$, the fidelity of the CN gate is gradually decreasing for larger dissipation rates until collapsing when the success rate is not exceeding 21%. Similarly, we illustrate in Fig. 5 the fidelity of the CCN gate against dissipation. The success of the realization of the CCN gate is gradually decreasing when the dissipation rates increase. For example, the success rate of the CCN realization is reaching levels of no more than 12% at dissipation rates greater than 0.2 for $\frac{\gamma _{1}}{\theta }$ and 0.1 for $\frac{\gamma }{\theta }$, which is significantly below the rates for the CN-gate. On the other hand, it is worthy to mention that the dissipation can be experimentally controlled through manipulating the Raman coupling with the lattice atoms. For instance, for weak Raman laser coupling with the lattice atoms, the dissipation is extremely small and can be neglected.

Finally, we point that recent numerical and experimental studies have demonstrated the feasibility of controlling dissipation in optical lattices with trapped ultracold atoms. For example, in Refs.^31,32^, it was shown that quantum gases can be manipulated to control dissipation and properly probing ultracold atoms. Also, in Ref.^33^, it was shown that a direct imaging of the vortex rings by phase slips can be used to control the microscopic dissipation dynamics of the ultracold atoms. Furthermore, in Refs.^34–36^, it has experimentally shown that the dissipative dynamics of the open quantum systems can be precisely controlled.

## Quantum algorithms

The CN and CNN gates combined with other gates (such as the one-atom gates of *i*PY, $\sqrt{-\text {I}_{2}}$ and Hadamard ($\mathcal {H}$) gates) can be employed to design novel schemes to realize quantum algorithms. Such algorithms can be used to solve quantum tasks including Simon, search, Shor, Fourier and Deutsch–Jozsa problems, and others. In quantum algorithms, the off-diagonal elements of the matrices that describe the CN and CCN gates are equal when dissipation is not taken into account. However,the diagonal elements are different once the dissipation is considered. While most previous reported schemes for quantum algorithms did not include dissipation, we will develop quantum algorithms taking dissipation into account.

### Novel algorithm to solve Simon’s problem

In this section, a novel algorithm will be developed using our proposed scheme to solve Simon problem (SP) to find a specific string or state (*s*).

#### Physical scheme

To realize Simon problem algorithm, a non-defective lattice is prepared as in the following: First, the lattice is prepared so that only single atom occupies a site, and all atoms are prepared in $|0\rangle = |\downarrow \rangle$ state. Second, the adjacent atoms in the $\wp _{1}$ and $\wp _{2}$ sites are prepared to contain *n*-atom. Third, an external Raman beams with proper Rabi frequencies and detuning are sent to $\wp _{1}$ and $\wp _{2}$ to couple them and generate the required quantum transitions. The steps to achieve the algorithm is detailed in the following:

- The atoms at $\wp _{1}$ and $\wp _{2}$ sites are prepared with state $|\phi _{1}\rangle =|0\rangle ^{\otimes n}_{1}|0\rangle ^{\otimes n}_{2}$.
- External laser beams are subjected to the non-interacting atom occupying $\wp _{1}$, and ZF effect is generated along *x*-axis. Consequently, the atom at evolution time = $\frac{\pi \hbar }{\sqrt{2}\zeta }$ can generate $\sqrt{-\text {I}_{2}}$ gate. Thus, the occupied atoms of $|0\rangle ^{\otimes n}_{1}$ inside $\wp _{1}$ sites undergo the transformations: $\mathfrak {I_{g}}=\sqrt{-\text {I}_{2}}\otimes \cdots \otimes \sqrt{-\text {I}_{2}}$, so $|\phi _{1}\rangle \Rightarrow |\phi _{2}\rangle =(\frac{-i}{\sqrt{2}})^{n}\sum _{u\in \{0,1\}^{n}}|u\rangle _{1}|0\rangle ^{\otimes n}_{2}$.
- External laser beams are sent to atoms occupying $\wp _{1}, \wp _{2}$ sites. First, the beams are tuned to be sent to non-interacting atom in $\wp _{1}, \wp _{2}$ to generate 1D ZF and 2D ZF effects. During the transition for each atom in the site, each atom can undergo the transformations and arise non-dissipative one-atom gates at specific pulses of time. Second, the beams are applied to couple the interacting atoms at $\wp _{1}, \wp _{2}$ sites and producing 1D SOC and 1D ZF. Consequently, each of the two interacting atoms can undergo dissipative transitions. Thus, one get $|\phi _{2}\rangle \Rightarrow |\phi _{3}\rangle =(\frac{-i}{\sqrt{2}})^{n} \sum _{\complement \in \{0,1\}^{n}}\sum _{q\in \{0,1\}^{n}}\mathfrak {D}^{q}_{\complement }(\gamma _{1},\gamma _{2})|q\rangle _{1}|\complement \rangle _{2}$, with $\mathfrak {D}^{q}_{\complement }$ denotes the damped coefficients due to applied dissipative gates across sites. Analytically, this means $B_{\mathfrak {F}}(\gamma _{1},\gamma _{2})|\phi _{2}\rangle = \sum _{\complement }\sum _{q}\mathfrak {D}^{q}_{\complement }(\gamma _{1},\gamma _{2})|q\rangle _{1}|\complement \rangle _{2}\langle u_{1},0^{\otimes n}_{2}|\phi _{2}\rangle =|\phi _{3}\rangle$.
- The coupled beams are adjusted so that each non-interacting atom of state $|q\rangle _{1}$ are producing ZF effect. It then follows that atoms undergo the transformation which generate $\sqrt{-\text {I}_{2}}^{\dagger }$ gate at time $\frac{3\pi \hbar }{\sqrt{2}\zeta }$. Consequently, the atoms of $|q\rangle _{1}$ will undergo the transformations $\sqrt{-\text {I}_{2}}^{\dagger }\otimes \cdots \otimes \sqrt{-\text {I}_{2}}^{\dagger }$, i.e. $|\phi _{3}\rangle \Rightarrow |\phi _{4}\rangle =\mathfrak {I^{\dagger }_{g}}|\phi _{3}\rangle =(\frac{1}{2})^{n}\sum _{\complement \in \{0,1\}^{n}}\sum _{w\in \{0,1\}^{n}} \mathfrak {\mathfrak {R}}^{w}_{\complement }(\gamma _{1},\gamma _{2})|w\rangle _{1}|\complement \rangle _{2}$, where the factors $\mathfrak {\mathfrak {R}}^{w}_{\complement }$ are dependent on $\mathfrak {D}^{q}_{\complement }$.
- Finally, the measurement of the atoms at $\wp _{1}$ and $\wp _{2}$ sites decides the required string *s*.

**Figure 6:**
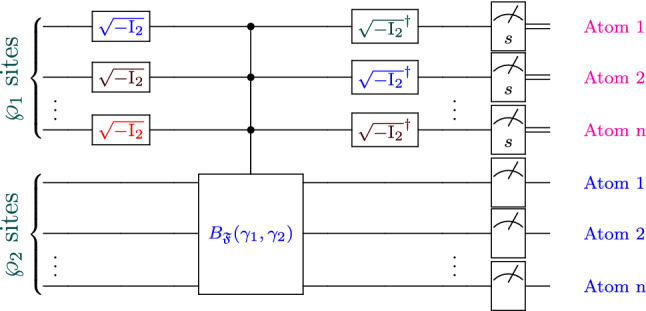
The proposed scheme to solve the Simon problem in the presence of dissipation, where $B_{\mathfrak {F}}(\gamma _{1},\gamma _{2})$ indicates the dissipative quantum circuits or black-boxes (oracles). For more details on the design of various types of $B_{\mathfrak {F}}(\gamma _{1},\gamma _{2})$, see Supplementary Information.

The proposed algorithm above can be represented schematically as in Fig. 6. According to the above-configurations, we can construct 95 new circuits of $B_{\mathfrak {F}}(\gamma _{1},\gamma _{2})$ across the confined atoms into $\wp _{1}$ and $\wp _{2}$ sites (see Supplementary Section S3A presented by Supplementary). The circuits of $B_{\mathfrak {F}}$ can represent various types of new oracles. After constructing these circuits, one needs to do a measurement or observation of the $\wp _{2}$ sites to know some queries.

#### Problem simulation with dissipation

Dissipation impact on Simon’s problem has not previously been studied. To take the dissipation into account, the first three steps of algorithm can be formulated by an unknown obfuscated circuit or oracle between two registers, *n* and $\jmath$, and we are required to compute a Boolean function $\mathfrak {F}: \{0,1\}^{n}\rightarrow \{0,1\}^{\jmath }$, with $\jmath \ge n$. The domain of $\mathfrak {F}$ (or the first register *n*) is equivalent to the atoms states at $\wp _{1}$ sites, while the co-domain of $\mathfrak {F}$ (or the second register $\jmath$) is equivalent to atoms states at $\wp _{2}$ sites. Our goal is to give some properties about $\mathfrak {F}$ to find *s*. Thus, for all $u_{j_{2}}, u_{j_{3}} \in \{0,1\}^{n}$ and $\mathfrak {F}(u_{j_{3}}) \in \{0,1\}^{\jmath }$, we observe that if $u_{1}\ne u_{2}\ne u_{3}\ne ...$ then $\mathfrak {F}(u_{1})=\mathfrak {F}(u_{2})=\mathfrak {F}(u_{3})=...$, where $u_{j_{2}}\equiv q$, $\mathfrak {F}(u_{j_{3}})\equiv \complement$ and $j_{2},j_{3}=1,2,...$ . Therefore, $\mathfrak {F}$ has only promised one property that for each $2^{n}$ inputs states of the first register have mapped to only one output state through the second register. This means that $\mathfrak {F}$ is a $2^{n}-\text {to}-1$ type function. As a result, due to the dissipation, the evaluation of $\mathfrak {F}$ that is known previously for individually being $1-\text {to}-1$ or $2-\text {to}-1$ will fail. Hence, the function $\mathfrak {F}$ can simultaneously possess the two classes through the dissipation. In other words, when there exists $s\in \{0,1\}^{n}$, we find that $\mathfrak {F}(u_{j_{3}})=\mathfrak {F}(u_{j_{2}})$ if and only if $u_{j_{3}}=s\oplus u_{j_{2}}$ and $u_{j_{3}}=u_{j_{2}}$, where $\oplus$ corresponds to addition modulo 2. So, we can simultaneously give a decision about the two cases of *s* $(s=0\,\, \text {and}\,\, s\ne 0, \,\,\text {where}\,\, 0\equiv 0^{\otimes n})$. Thus, after performing the algorithm to solve the problem, if $\mathfrak {F}(u_{j_{3}})=\eta _{1}$, the probability to observe each string of *w* to determine, $s=0$, will be given by: $\text {Pr}_{(s=0)}=\frac{|(-1)^{q\cdot w}\mathfrak {D}^{q}_{\eta _{1}}(\gamma _{1},\gamma _{2})|^{2}}{2^{2n}} =\frac{\sum ^{2^{n}}_{k_{1}=1}|\mathfrak {D}^{q}_{k_{1}}(\gamma _{1},\gamma _{2})|^{2}}{2^{2n}}$. For two various possible states of the first register and any state $\eta _{2}\in \complement$, if $\mathfrak {F}(u_{3})=\eta _{2}=\mathfrak {F}(u_{5})$, the probability to measure this part of the first register to decide, $s\ne 0$, will be given by: $\text {Pr}_{(s\ne 0)}=\frac{|(-1)^{u_{3}\cdot w}\mathfrak {D}^{u_{3}}_{\eta _{2}}(\gamma _{1},\gamma _{2})+(-1)^{u_{5}\cdot w}\mathfrak {D}^{u_{5}}_{\eta _{2}}(\gamma _{1},\gamma _{2})|^{2}}{2^{2n}} =\frac{\sum _{k_{1}}\{|\mathfrak {D}^{u_{3}}_{k_{1}}(\gamma _{1},\gamma _{2})|^{2} +|\mathfrak {D}^{u_{5}}_{k_{1}}(\gamma _{1},\gamma _{2})|^{2}\}}{2^{2n}}$. However, we find that, $\mathfrak {D}^{j_{5}}_{k_{1}}(0,0)=1$ for any $j_{5}$. So, the values of probabilities to observe *w* for deciding *s* are $\frac{1}{2^{n}}$ and $\frac{1}{2^{n-1}}$ of the two cases of $\mathfrak {F}$. Thus, the absence case of dissipation coincides with the previous studies of Simon problem.

### Deciding of string

In the following, we define the procedures needed to find string *s*. After performing the first three steps of the proposed algorithm above, each $2^{n}$ various states through $\wp _{1}$ sites correspond to only one state through $\wp _{2}$ sites. So, every two various states across $\wp _{1}$ with only one state through $\wp _{2}$ can be demonstrated by the superposition, $(\frac{-i}{\sqrt{2}})^{n}\{\mathfrak {D}^{u_{1}}_{\eta _{2}}(\gamma _{1},\gamma _{2})|u_{1}\rangle _{1} +\mathfrak {D}^{u_{2}}_{\eta _{2}}(\gamma _{1},\gamma _{2})|u_{2}\rangle _{1}\}|\eta _{2}\rangle _{2}$. We predict that the coefficients for any two different states of the first register, say $\mathfrak {D}^{u_{1}}_{\eta _{2}}$ and $\mathfrak {D}^{u_{2}}_{\eta _{2}}$, can be identical after performing specific $B_{\mathfrak {F}}(\gamma _{1},\gamma _{2})$. Thus, after applying $\mathfrak {I^{\dagger }_{g}}$ gates to the register of $\wp _{1}$-site, such a superposition will become $\frac{1}{2^{n}}\sum _{w}(-1)^{u_{1}\cdot w}\{\mathfrak {D}^{u_{1}}_{\eta _{2}}(\gamma _{1},\gamma _{2})+(-1)^{s\cdot w}\mathfrak {D}^{u_{2}}_{\eta _{2}}(\gamma _{1},\gamma _{2})\}|w\rangle _{1}|\eta _{2}\rangle _{2}$, where $u_{2}\cdot w=(s\oplus u_{1})\cdot w$, $w=\{w_{1},w_{2},...,w_{2^{n}}\}\in \{0,1\}^{n}$ and $w_{1}\ne ...\ne w_{2^{n}}$. To be able to perform any measurement of the first register, $s\cdot w=0$ must be satisfied, to avoid reaching zero due to the equality of $\mathfrak {D}^{q}_{\complement }$ coefficients. Hence, the measurement for each element through the first register always results in some state of $w_{l}$ that satisfies the constraint $s\cdot w_{l}=0$ (modulo 2), $l=1,2,...,2^{n}$. So, after the measurement for each state of *w*, we find that the linearly independent equations of the form: $s\cdot w_{1}=0$, $s\cdot w_{2}=0$, ..., $s\cdot w_{l}=0$. We know that $s\cdot w_{l}=s_{0}w_{l0}\oplus s_{1}w_{l1}\oplus \cdots \oplus s_{j_{1}}w_{lj_{1}}$, where $s_{0}$,..., $s_{j_{1}}$ and $w_{l0}$,..., $w_{lj_{1}}$ are the bits of strings *s* and $w_{l}$, respectively. One solution of such equations is the trivial solution, say $w_{1}=0$, therefore, there are $l-1$ non-trivial linearly independent solutions that are sufficing to decide $s=0$ and $s\ne 0$. Thus, it is easy to show that $\Xi (n)$ queries to the black-box in a polynomial-time can be covered to compute the different values of *s*. In other words, following the performance of the steps of the algorithm, we need 2, 3, 4 non-trivial solutions and so on through the first register (i.e., we need 2, 3, 4 various states of *w*) to determine *s* of the 2-, 3-, 4-atom algorithm and so on, respectively. For more details to decide *s* analytically (see Supplementary Part 3B shown by supplementary).

Finally, we note that our proposed algorithm to solve Simon problem can be applied to many other different tasks. This include studying machine learning models^37,38^, and observing the insecurity of commonly-utilized cryptographic symmetric-key primitives^39^. Furthermore, our algorithm can also be used to study quantum communication systems.

## Conclusion

We proposed and thoroughly investigated the scheme of employing trapped ultracold atoms in optical lattice to function as viable platform for quantum CN and CCN gates. The trapped ultracold atoms are subjected to spin-orbital coupling and Zeeman field splitting. A non-Hermitian Hamiltonian is derived considering weak tunneling and strong interaction strength (in comparison to the SOC and ZF effects). The obtained Hamiltonian framework encompasses the Heisenberg model, 3D anisotropic Dzyaloshinskii–Moriya interactions, and dissipation. Consequently, CN and CCN gates have been developed and evaluated. It is shown that the proposed scheme provides an efficient gates’ performance as compared to previously reported gates. This includes the number of gates (and processing time) required to realize the CN and CCN gates circuits. Furthermore, quantum algorithms have been evaluated using the proposed scheme and new oracles (and deciding string algorithm) were developed. Finally, we note that our developed circuits can also be applied to many other problems, such as search, Shor, Fourier and Deutsch–Jozsa problems, just to mention a few examples.

## Supplementary Information

Supplementary Information.

## Data Availability

The datasets generated during and/or analysed during the current study are available from the corresponding author on reasonable request.
