# Supplementary material for: Efficient quantum gates and algorithms in an engineered optical lattice
Source: Sci Rep. 2021 Jul 28;11:15402. doi: 10.1038/s41598-021-94929-4 (PMC8319201; doi:10.1038/s41598-021-94929-4)
Supplement: Supplementary file 1 — Supplementary Information. [file 41598_2021_94929_MOESM1_ESM.pdf]

Supplementary Material: Efficient Quantum Gates and Algorithms in an Engineered Optical Lattice

A. H. HOMID, M. ABDEL-ATY, M. QASYMEH, AND H. ELEUCH

This document provides supplementary information to "Efficient Quantum Gates and Algorithms in an Engineered Optical Lattice".

1. STRUCTURE OF THE PHYSICAL SYSTEM

A. Brief review of the experimental-setup of engineered lattice

Optical lattices, whcich are artificial crystals of light, are considered to be one of the key potential techniques for quantum simulation of matters. The optical lattice (or crystal) can be created by applying counter-propagating laser lights to ultracold atomic gas at low temperature, see Fig.(S1a). The laser beams produce a lattice-like potential that is with periodic spatial dependence. In this work, we consider the laser beams applied from four directions, and thus, a square

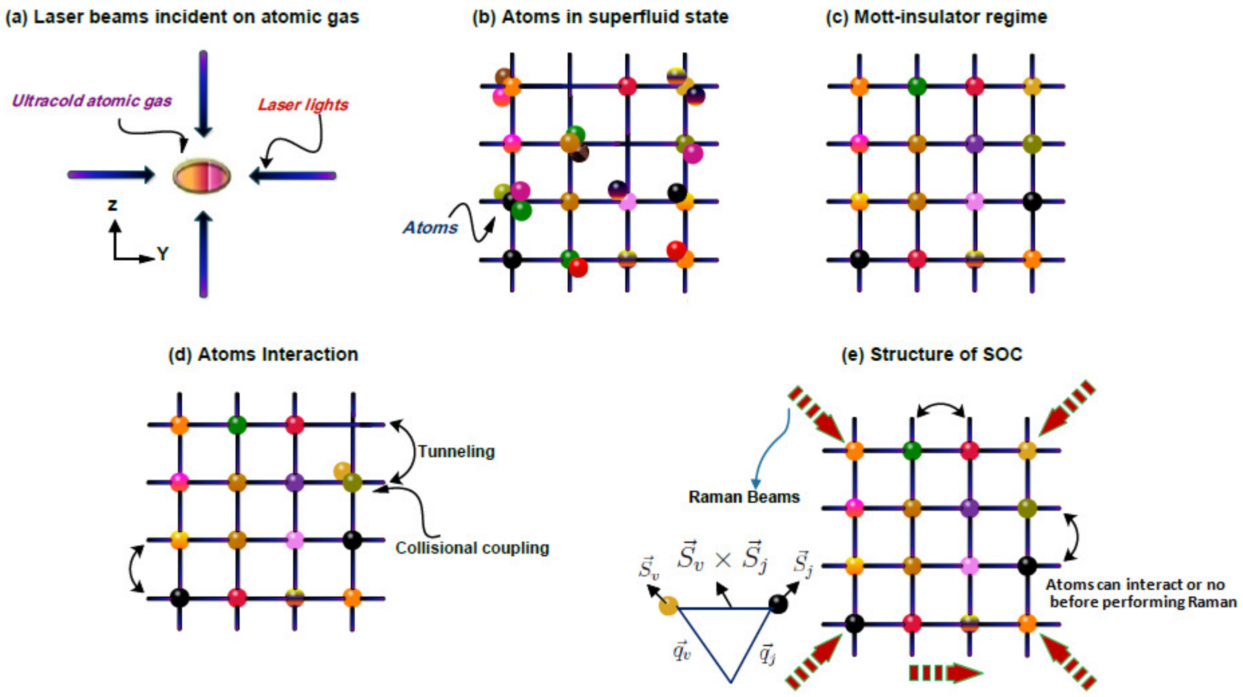

Fig. S1. Simplified layout of the experimental-setup to create an artificial square lattice or crystal, and then the mott-insulator phase. Following, it performs the Raman beams to this lattice.

two dimensional (2D) lattice is created, see Fig.(S1a). Such lattice can be conceived ideal with no impurities (or vibrations) by employing laser beams with highly quality and stability. The periodic potential is given by: $L(y,z) = \sum_{r=y,z} L_0 \sin^2(\frac{2\pi r}{\lambda_l})$, where L_0 is the maximum potential depth of the laser and $\lambda_l = \frac{c}{\nu_l}$ refers to the laser wavelength, c is the speed of light and ν_l is the photon frequency. Consequently, the atoms are trapped in a yz-plane and frozen along the

x-direction. Here $r_e = \frac{(\hbar\Lambda_l)^2}{2m}$ is the recoil energy, m is the mass of single atom and $\Lambda_l = 2\pi/\lambda_l$ is the laser wavevector. $L(y,z)$ is considered sufficiently strong, and λ_l is twice the lattice distance, so that no atoms resonance is occurring. It then follows that the atoms do not absorb the laser photons. In this case, the atoms can maintain their temperature for some time. The atoms in the lattice are with superfluid state at low temperatures, and can be freely moving all around the lattices sites. That is due to the strong intensities of the laser and the sufficient depth of the optical lattice potential. Such depth can be tuned by changing the laser power. Also, for the case of the superfluid state, four possibilities are corresponding to each site, being empty state, having an up or down spin state ($|\uparrow\rangle, |\downarrow\rangle$) or being doubly occupied state ($|\uparrow\downarrow\rangle$), see Fig.(S1b). The atoms in the optical lattice can obey a deep mott-insulator phase in which each site accommodates one atom only [1], see Fig.(S1c). In other words, the lattice atoms density in mott-insulator regime is low enough so that only one atom exists at a site, i.e. $\langle\hat{n}_{v\uparrow}\rangle + \langle\hat{n}_{v\downarrow}\rangle \simeq 1$. Here, $\hat{n}_v$ denotes the atoms number operator, which is 0 or 1 for the fermions atoms and natural number for the bosons atoms. The atoms in the mott-insulator case cannot move freely, where they are confined in a potential minima. Hence, the spin degree of freedom is activated, and the mass degree of freedom is frozen. Consequently, each site is occupied by one atom that has two relevant internal states $|\downarrow\rangle, |\uparrow\rangle$. Importantly, this is an ideal quantum bit realization. The interactions between atoms in various sites occur through virtual transitions. These transitions are dictated by the tunneling coupling and the collisional coupling, see Fig.(S1d), and play a significant role in the quantum computation. These virtual interactions in the 2D (yz-plane) engineered lattice, that contains $\wp$ sites, can be described by the following Hamiltonian [2]:

$$\hat{H}_{op} = \frac{1}{2}\sum_{v=1}^{\wp}\sum_{k=\uparrow,\downarrow}\left[\Omega_{kk}\hat{n}_{vk}(\hat{n}_{vk}-1) + \Omega_{k\bar{k}}\,\hat{n}_{vk}\hat{n}_{v\bar{k}} - 2\mu_k\hat{n}_{vk}\right] - \sum_{v,j=1}^{\wp}\sum_{k=\uparrow,\downarrow}\left(g_k\hat{a}^{\dagger}_{vk}\hat{a}_{jk} + g^{*}_{k}\hat{a}^{\dagger}_{jk}\hat{a}_{vk}\right), \tag{S1}$$

where $\hat{a}_{vk}$ refers to the lowering operator of bosonic (or fermionic) atom of spin state k at site v, $\hat{n}_{v\uparrow} = \hat{a}^{\dagger}_{v\uparrow}\hat{a}_{v\uparrow}$ is the number operator for the boson (or fermion) atoms, $\wp$ refers to the sites number, μ_k indicates the chemical potential of the interacting atoms, $g_k = \int dr\varrho^{*}_{vk}(r)[\hat{p}^2/2m + L(r)]\varrho_{jk}(r)$ denotes the tunneling matrix element between adjacent sites v, j, ϱ_{jk} is the Wannier state, $\vec{p} = (\hat{p}_y, \hat{p}_z)$ is the momentum operator vector, and $\Omega_{kk'} = (4\pi\ell_s\hbar^2/m)\int dr|\varrho_{vk}|^2|\varrho_{vk'}|^2$ expresses the interaction strength (or the repulsion) of two atoms of an individual site between species $k, k' = (\uparrow,\downarrow)$, and ℓ_s is the scattering length between the atoms. Given a short-range interactions, compared to the space of lattice, the interaction strength between atoms can be described by $\Omega_{kk'}$. Here, the first term of Eq.(S1) (i.e., $\hat{n}_{vk}(\hat{n}_{vk}-1)$) vanishes for fermion atoms. In (S1), we note that, $g^{*}_{\uparrow} = g_{\downarrow}$, $g^{*}_{\downarrow} = g_{\uparrow}$, $\mu_{\uparrow(\downarrow)} = +(-)\mu$, $\Omega_{\uparrow\bar{\uparrow}} = \Omega_{\uparrow\downarrow}$, $\Omega_{\downarrow\bar{\downarrow}} = \Omega_{\downarrow\uparrow}$, $\Omega_{\uparrow\downarrow} = \Omega_{\downarrow\uparrow}$, $\bar{k} = -k$, $\hat{a}_{-v\uparrow} = -\hat{a}_{v\downarrow}$ and $\hat{a}_{-v\downarrow} = -\hat{a}_{v\uparrow}$.

B. Synthesis of SOC and ZF mechanisms inside lattice

In this section, the Hamiltonian of 2D crystal lattice, that incorporates spin-orbit coupling (SOC) and 2D Zeeman field (ZF) mechanisms, is developed . Herein, the SOC mechanism indicates the intrinsic interaction between the neutral atoms spin and its motion. Experimentally, the SOC and ZF phenomena are realized by applying external Raman laser to the confined atoms in 2D lattice (yz-plane), see Fig.(S1e). One of the components of 2D ZF is perpendicular to SOC, and the other is parallel to SOC. Such experiment indicate that the Raman beams can be employed to dress atoms with spin-dependent momentums [3]. Here, each lattice site is a sample of two hyperfine ground states that can be coupled with an excited state by utilizing Raman beams with proper Rabi frequencies [4–6]. Three to thirteen Raman lasers of uniform plane waves are used for generating the required SOC and ZF dynamics. The two degenerate dark states in Refs.[4–6] can be defined here in the form of $\{|\uparrow\rangle, |\downarrow\rangle\}$ states. Using the same procedure in [4–6], the coefficients of the subspace of the dark states can be determined via the Raman laser parameters to obtain the effective low-energy Hamiltonian. Upon projecting into the subspace of dark states, the resulting Hamiltonian is given by: $\hat{H}_{mec} = \alpha_1(\hat{p}_y\hat{\sigma}_z - \hat{p}_z\hat{\sigma}_y) + \alpha_2\hat{\sigma}_x - \alpha_3\hat{\sigma}_y$, where $\vec{\sigma} = (\hat{\sigma}_x, \hat{\sigma}_y, \hat{\sigma}_z)$ is the vector of Pauli matrices, and $\alpha_1 \sim \frac{\hbar\Lambda_R}{2m\sqrt{3}}$, $\alpha_2 \sim \frac{3\hbar\omega_0^2}{\Delta_R}$ and $\alpha_3 \sim \frac{\hbar\varpi_p}{\Delta_R}$ denote the strengths of SOC, perpendicular ZF and parallel ZF, respectively, [4–6]. Here, Λ_R is the Raman laser wavevector, ω_0 is the Rabi frequency magnitude, and Δ_R is the detuning of the Raman laser. The parameter ϖ_p is

a known experimental parameter [4, 6]. In the most cases, Λ_l analogous with Λ_R (i.e., $\Lambda_R \simeq \Lambda_l$). Thus, the system $\hat{H}_{mec}$ in the continuous space is described as the pairing between the spin-flip and the neighboring sites hopping, as in the following:

$$\begin{aligned}\hat{H}_{soz} &= \alpha_1 \int dr\{\hat{\phi}^\dagger(r)(\hat{p}_y\hat{\sigma}_z - \hat{p}_z\hat{\sigma}_y)\hat{\phi}(r) + h.c.\} \\ &+\alpha_2 \int dr\{\hat{\phi}^\dagger(r)\hat{\sigma}_x\hat{\phi}(r) - h.c.\} - \alpha_3 \int dr\{\hat{\phi}^\dagger(r)\hat{\sigma}_y\hat{\phi}(r) + h.c.\},\end{aligned} \tag{S2}$$

where $\hat{\phi}(r)$ can be approximate in case of single-band by: $\sum_{j,k} \hat{a}_{jk}\varrho_{jk}(r)$. Hence, the Hamiltonian for any two-site v and j in the optical lattice can be given by:

$$\begin{aligned}\hat{H}_{SO-Z} &= \sum_{v,j=1}^{\wp} \sum_{k=\uparrow,\downarrow} \big[\{i\hat{a}^\dagger_{vk}(\hat{e}_x \cdot (\vec{\zeta} \times \vec{\varepsilon}_{vj}))\hat{a}_{jk} - h.c.\} + \zeta_2\{\hat{a}^\dagger_{vk}\hat{\sigma}_x\hat{a}_{jk} - \hat{a}^\dagger_{jk}\hat{\sigma}_x\hat{a}_{vk}\} \\ &-\zeta_3\{\hat{a}^\dagger_{vk}\hat{\sigma}_y\hat{a}_{jk} + \hat{a}^\dagger_{jk}\hat{\sigma}_y\hat{a}_{vk}\}\big],\end{aligned} \tag{S3}$$

where $\hat{e}_x = (1, 0, 0)$ is x-unit vector, $\vec{\zeta} = (\hat{\sigma}_x, \zeta_1^z\hat{\sigma}_y, \zeta_1^y\hat{\sigma}_z)$ and $\vec{\varepsilon}_{vj} = (0, 1, 1)$ is the vector from a site j at position q_j to a site v at position q_v.m The values of $\zeta_1^r = \hbar\alpha_1 \int dr\varrho^*_{vk}(r)\frac{\partial}{\partial r}\varrho_{jk}(r)$, $\zeta_2 = \alpha_2 \int dr\varrho^*_{vk}(r)\varrho_{jk}(r)$ and $\zeta_3 = \alpha_3 \int dr\varrho^*_{vk}(r)\varrho_{jk}(r)$ can be tuned by employing the coherent destructive tunneling methods [10]. Here, we assume that the parameters ζ_1^y, ζ_1^z and ζ_2 are much less than $\Omega_{kk'}$. Using the systems (S1,S3), the Hamiltonian of the spin-$\frac{1}{2}$ fermions (or bosons) atoms is given by:

$$\hat{H}_{inc} = \hat{H}_{op} + \hat{H}_{SO-Z} = \hat{H}_1 + \hat{H}_0, \tag{S4}$$

where $\hat{H}_0$ and $\hat{H}_1$ are given by:

$$\begin{aligned}\hat{H}_1 &= \sum_{v,j=1}^{\wp} \sum_{k=\uparrow,\downarrow} \{ -(g_k\hat{a}^\dagger_{vk}\hat{a}_{jk} + h.c.) + [i\hat{a}^\dagger_{vk}(\hat{e}_x \cdot (\vec{\zeta} \times \vec{\varepsilon}_{vj}))\hat{a}_{jk} - h.c.] \\ &+\zeta_2[\hat{a}^\dagger_{vk}\hat{\sigma}_x\hat{a}_{jk} - \hat{a}^\dagger_{jk}\hat{\sigma}_x\hat{a}_{vk}] - \zeta_3[\hat{a}^\dagger_{vk}\hat{\sigma}_y\hat{a}_{jk} + \hat{a}^\dagger_{jk}\hat{\sigma}_y\hat{a}_{vk}]\}. \\ \hat{H}_0 &= \sum_{v,k}\{\frac{1}{2}\Omega_{kk}\hat{n}_{vk}(\hat{n}_{vk} - 1) + \frac{1}{2}\Omega_{k\bar{k}}\,\hat{n}_{vk}\hat{n}_{v\bar{k}} - \mu_k\hat{n}_{vk}\}.\end{aligned}$$

For each atom occupying a site, say v, that is governed by the system (S4), the possible states (for pseudo-spin basis) can be given by: $|\uparrow\rangle = |\hat{n}_{v\uparrow} = 1; \hat{n}_{v\downarrow} = 0\rangle$ and $|\downarrow\rangle = |\hat{n}_{v\downarrow} = 1; \hat{n}_{v\uparrow} = 0\rangle$. We also have: $\hat{a}_{v\uparrow}|\downarrow\rangle = 0|\hat{n}_{v\downarrow} = 1; \hat{n}_{v\uparrow} = -1\rangle = 0$, $\hat{a}_{v\uparrow}|\uparrow\rangle = |\hat{n}_{v\uparrow} = 0; \hat{n}_{v\downarrow} = 0\rangle = |0\rangle$, $\hat{a}^\dagger_{v\uparrow}|\downarrow\rangle = |\hat{n}_{v\downarrow} = 1; \hat{n}_{v\uparrow} = 1\rangle = |\uparrow\downarrow\rangle$, $\hat{a}^\dagger_{v\uparrow}|\uparrow\rangle = \sqrt{2}|\hat{n}_{v\uparrow} = 2; \hat{n}_{v\downarrow} = 0\rangle = \sqrt{2}|\uparrow\uparrow\rangle$, $\hat{a}_{v\downarrow}|\downarrow\rangle = |0\rangle$, $\hat{a}_{v\downarrow}|\uparrow\rangle = 0$, $\hat{a}^\dagger_{v\downarrow}|\downarrow\rangle = \sqrt{2}|\downarrow\downarrow\rangle$ and $\hat{a}^\dagger_{v\downarrow}|\uparrow\rangle = |\uparrow\downarrow\rangle$. Thus, the virtual possible excited states are six states for bosons, and two states for fermions, see Fig.(S2). The states of the bosonic atoms are $|\uparrow\uparrow; 0\rangle$, $|0; \uparrow\uparrow\rangle$, $|\downarrow\downarrow; 0\rangle$, $|0; \downarrow\downarrow\rangle$, $|\uparrow\downarrow; 0\rangle$ and $|0; \uparrow\downarrow\rangle$, while the states of the fermionic atoms are $|\uparrow\downarrow; 0\rangle$ and $|0; \uparrow\downarrow\rangle$.

C. Formulation of non-Hermitian Hamiltonian

Typically, in engineered lattices, a number of two or more atoms are located at the same site. Hence, significant collision coupling can take place. However, for weak tunneling transitions, the average number of atoms at a site can be conceived constant. Consequently, on having the interaction strength between any two atoms with different internal states ($|\uparrow\rangle$ and $|\downarrow\rangle$) significantly larger than the SOC and ZF effects, virtual transitions between levels take place and the degeneracy in spin configurations can be ignored. Accordingly, this degeneracy can be removed and the system evolution can be described by an effective Hamiltonian. To this end, we apply a non-unitary transformation to the open quantum systems Hamiltonian (S4). The action of this transformation is inspired from the concept of the Schrieffer-Wolff transformation [7]. The proposed transformation includes a non-Hermitian generator, $\hat{O}_\pm$, so the system (S4) in the new gauge reads:

$$\hat{H} = e^{(\hat{O}_\pm)}\,\hat{H}_{inc}\,e^{(-\hat{O}_\pm)} = \hat{H}_0 + \hat{H}_1 + \frac{1}{1!}[\hat{O}_\pm, \hat{H}_0 + \hat{H}_1] + \frac{1}{2!}[\hat{O}_\pm, [\hat{O}_\pm, \hat{H}_0 + \hat{H}_1]] + \dots .$$

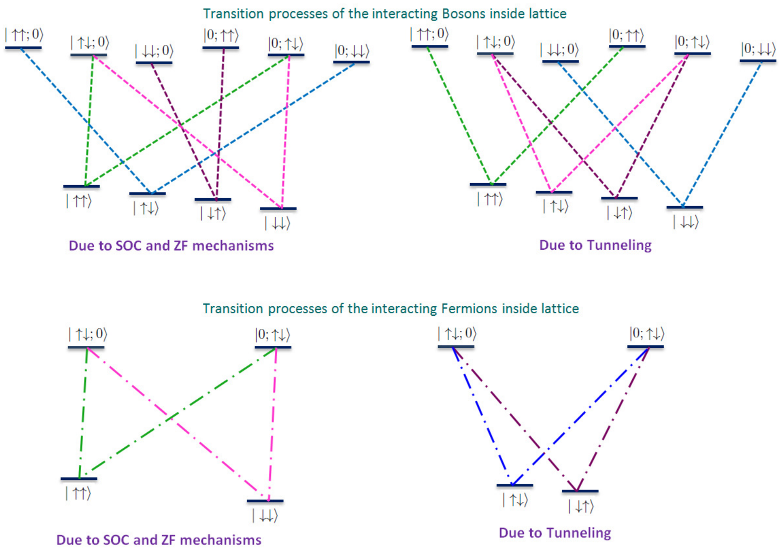

Fig. S2. The available excited states of atoms in the engineered lattice that can obtain by acting on the ground states ($|\uparrow\uparrow\rangle, |\uparrow\downarrow\rangle, |\downarrow\uparrow\rangle, |\downarrow\downarrow\rangle$) across the system (S4).

Here $\hat{O}_+$ is for $\Omega_{\uparrow\uparrow} = \Omega_{\downarrow\downarrow} = \Omega_{\uparrow\downarrow}$ for boson atoms, and $\hat{O}_-$ is for $\Omega_{\uparrow\uparrow}, \Omega_{\downarrow\downarrow} \to \infty$ for fermion atoms. These are defined as in the following:

$$\hat{O}_{\pm} = \sum_{v,j,k} \{ \frac{\hat{n}_{v\bar{k}}}{(\mu_k \pm \Omega_{\uparrow\downarrow})} + \frac{(1-\hat{n}_{v\bar{k}})}{\mu_k} \} \{ [(g_k - i\eta_k)\hat{a}^{\dagger}_{vk}\hat{a}_{jk} - h.c.] + [(\varepsilon_k + \zeta_2)\hat{a}^{\dagger}_{v\bar{k}}\hat{a}_{jk} - h.c.] \},$$

where $\varepsilon_{\uparrow(\downarrow)} = -(+)\zeta_4$, $\zeta_4 = e^{i\theta_1}\sqrt{(\zeta_1^z)^2 + \zeta_3^2}$, $\theta_1 = \arctan(\frac{\zeta_3}{\zeta_1^z})$, $\varepsilon^*_{\uparrow} = \varepsilon_{\downarrow}$, $\varepsilon^*_{\downarrow} = \varepsilon_{\uparrow}$, $\eta_{\uparrow(\downarrow)} = -(+)\zeta_1^y$, $\zeta_2^* = -\zeta_2$, and $\eta_k^* = \eta_k$. The generator satisfies $[\hat{O}_{\pm}, \hat{H}_0] = -\hat{H}_1$, and thus:

$$\hat{H} \simeq \hat{H}_0 + \frac{1}{2}[\hat{O}_{\pm}, \hat{H}_1], \tag{S5}$$

where $[\hat{O}_{\pm}, [\hat{O}_{\pm}, \hat{H}_1]] \cong 0$ for the case of g_k, $\zeta_1^{z(y)}$, $\zeta_2, \zeta_3 \ll \Omega_{\uparrow\downarrow}$. On calculating the required commutators, and using the iso-spin operators ($\vec{S}_v$), the Hamiltonian (S5) can be expressed as in the following:

$$\begin{aligned}\hat{H} \simeq & \sum_{v,j=1}^{\wp} \{ J_x \hat{S}_v^x \hat{S}_j^x + J_y \hat{S}_v^y \hat{S}_j^y + J_z \hat{S}_v^z \hat{S}_j^z + D_x(\hat{S}_v^y \hat{S}_j^z - \hat{S}_v^z \hat{S}_j^y) + D_y(\hat{S}_v^z \hat{S}_j^x - \hat{S}_v^x \hat{S}_j^z) \\ & + D_z(\hat{S}_v^x \hat{S}_j^y - \hat{S}_v^y \hat{S}_j^x) \} - \sum_{v=1}^{\wp} f_z \hat{S}_v^z - i\gamma_1 \sum_{v=1}^{\wp} \hat{S}_v^+ \hat{S}_v^- - i\gamma_2 \sum_{v,j=1}^{\wp} (\hat{S}_v^+ \hat{S}_j^- + \hat{S}_v^- \hat{S}_j^+) \\ & - 4i\gamma_2 \sum_{v \neq j}^{\wp} (\hat{S}_v^+ \hat{S}_j^-)(\hat{S}_j^+ \hat{S}_v^-) + \aleph \hat{I},\end{aligned} \tag{S6}$$

where $\vec{S}_v = \{\hat{S}_v^x, \hat{S}_v^y, \hat{S}_v^z\} = \frac{1}{2}\sum_{k,k'} \hat{a}^{\dagger}_{vk}\hat{\sigma}_{kk'}\hat{a}_{vk'}$, $\hat{\sigma}_{kk'}$ indicate the elements of Pauli matrices for each corresponding spin operator, $J_x = J_y = \frac{\pm\Omega_{\uparrow\downarrow}[2g_{\uparrow}g_{\downarrow} - 2(\zeta_1^y)^2]}{\Omega^2}$, $J_z = \{\frac{\pm\Omega_{\uparrow\downarrow}[-(g_{\uparrow}+g_{\downarrow})^2 + (2\zeta_1^y)^2 + 4\zeta_4^2 + 4\zeta_2^2]}{\Omega^2} + \frac{4\zeta_4\zeta_2(\Omega_{\uparrow\downarrow}^2 + \mu^2)}{\mu\Omega^2}\}$, $D_x = \{\frac{4(\pm\Omega_{\uparrow\downarrow}\zeta_1^y\zeta_4 + \mu\zeta_2)}{\Omega^2} + \frac{4\zeta_1^y\zeta_2}{\mu}\}$, $D_y = [\frac{2(\pm\Omega_{\uparrow\downarrow}\zeta_2 + \mu\zeta_4)}{\Omega^2} + \frac{2\zeta_4}{\mu}](g_{\uparrow} - g_{\downarrow})$, $D_z = \frac{\pm 2\Omega_{\uparrow\downarrow}\zeta_1^y(g_{\uparrow}+g_{\downarrow})}{\Omega^2}$, $f_z = \{\frac{[\mp 8\Omega_{\uparrow\downarrow}\zeta_4\zeta_2 - 4\mu(\zeta_4^2+\zeta_2^2)]}{\Omega^2} + \frac{\Omega_{\uparrow\downarrow}^2[-g_{\uparrow}^2 - g_{\downarrow}^2 + 2(\zeta_1^y)^2]}{\mu\Omega^2} + 2\mu\}$, $\gamma_1 = \frac{\pm 2\Omega_{\uparrow\downarrow}\zeta_1^y(g_{\uparrow}+g_{\downarrow})}{\Omega^2}$, $\gamma_2 = \zeta_1^y(g_{\uparrow} + g_{\downarrow})[\frac{\mu}{\Omega^2} + \frac{1}{\mu}]$, $\Omega^2 = (\Omega_{\uparrow\downarrow}^2 - \mu^2)$, and $\aleph = \{\frac{\mp\Omega_{\uparrow\downarrow}[(g_{\uparrow}-g_{\downarrow})^2 + 4\zeta_4^2 + 4\zeta_2^2]}{4\Omega^2} - \frac{2\zeta_4\zeta_2\Omega_{\uparrow\downarrow}^2}{\mu\Omega^2} + i\zeta_1^y(g_{\uparrow} + g_{\downarrow})[\frac{(\mu\pm\Omega_{\uparrow\downarrow})}{\Omega^2} + \frac{1}{\mu}]\}$.

The Hamiltonian (S6) describes both bosons and fermions atoms, where the positive (negative)

sign of $\pm\Omega_{\uparrow\downarrow}$ is for bosons (fermions) atoms. The values of f_z and J_z coefficients are not equal as shown in previous studies [8, 9]. Also, in the case of $\Omega_{\uparrow\downarrow} = 2\mu$, we note that $\gamma_1 = \gamma_2$. The term $\Xi_x(\hat{S}_v^x + \hat{S}_j^x)$, with $\Xi_x = [(\zeta_2\Omega^2 \pm \mu\zeta_4\Omega_{\uparrow\downarrow})(g_\uparrow - g_\downarrow)/\mu\Omega^2]$, in the system (S6) is produced through the generation of Dzyaloshinskii-Moriya interaction (DMI). This term has no effect on the following results of our work and can be omitted.

2. ENGINEERING CONTROL TO REALIZE QUANTUM GATES

The SOC and ZF mechanisms can be engineered to implement quantum gates. The SOC effect can be used to optimize the gates' cost. This approach of using the SOC effect for cost optimization has not been previously considered.

A. Dissipative one-atom gates

In this sucbsection, we consider one-atom gates with dissipation.Consequently, when the tunneling and interaction between atoms are vanishing ($\Omega_{kk'} = 0$, $g_k = 0$ and $\vec{\varepsilon}_{vj} = \vec{0}$), the Hamiltonian in Eq.(S4), for Raman lasers coupled to single-atom a site v, is given by:

$$\hat{H}_v = \frac{\zeta_x}{2}(\hat{a}^\dagger_{v\uparrow}\hat{a}_{v\downarrow} + \hat{a}^\dagger_{v\downarrow}\hat{a}_{v\uparrow}) - \frac{\zeta_y}{2}(-i\hat{a}^\dagger_{v\uparrow}\hat{a}_{v\downarrow} + i\hat{a}^\dagger_{v\downarrow}\hat{a}_{v\uparrow}) - \frac{(\zeta_\mu + i\vee_y)}{2}(\hat{a}^\dagger_{v\uparrow}\hat{a}_{v\uparrow} - \hat{a}^\dagger_{v\downarrow}\hat{a}_{v\downarrow}), \quad \text{(S7)}$$

where $\zeta_x = 2\alpha_2 \int dr\varrho^*_{vk}\varrho_{vk}$, $\zeta_y = e^{-i\theta_2}\sqrt{\vee_z^2 + \zeta_\kappa^2}$, $\zeta_\mu = 2\mu_0 \int dr\varrho^*_{vk}\varrho_{vk}$, $\zeta_\kappa = 2\alpha_3 \int dr\varrho^*_{vk}\varrho_{vk}$, $\vee_y = 2\hbar\alpha_1 \int dy\varrho^*_{vk}(y)\frac{\partial}{\partial y}\varrho_{vk}(y)$, $\vee_z = 2\hbar\alpha_1 \int dz\varrho^*_{vk}(z)\frac{\partial}{\partial z}\varrho_{vk}(z)$, and $\theta_2 = \arctan(\vee_z/\zeta_\kappa)$. The chemical potential in the mott-insulator phase and non-interacting atoms represented by $\mu_\uparrow = \mu_0$ and $\mu_\downarrow = -\mu_0$. Accordingly, the system (S7) can be introduced in term of the iso-spin operators ($\hat{\sigma}_v^x = \hat{a}^\dagger_{v\uparrow}\hat{a}_{v\downarrow} + \hat{a}^\dagger_{v\downarrow}\hat{a}_{v\uparrow}$, $\hat{\sigma}_v^y = -i\hat{a}^\dagger_{v\uparrow}\hat{a}_{v\downarrow} + i\hat{a}^\dagger_{v\downarrow}\hat{a}_{v\uparrow}$ and $\hat{\sigma}_v^z = \hat{a}^\dagger_{v\uparrow}\hat{a}_{v\uparrow} - \hat{a}^\dagger_{v\downarrow}\hat{a}_{v\downarrow}$), yielding:

$$\hat{H}_v^s = \zeta_x\hat{S}_v^x - \zeta_y\hat{S}_v^y - \zeta_\mu\hat{S}_v^z - i\gamma\hat{S}_v^+\hat{S}_v^-, \quad \text{(S8)}$$

where $\gamma = \vee_y$ is the damping (or dissipating) parameter of the one-atom system. The Hamiltonian (S8) is non-Hermitian. The dissipation effect contributes on state $|\uparrow\rangle$ decaying to the state $|\downarrow\rangle$. This dissipation leads to imperfect achievement of the gates. For the system (S8), the constant term $\frac{i\vee_y}{2}$ does not include any damping and has no effect during the gates' realization. Hence, this term is omitted for the forthcoming analysis.

A.1. Proposed one-atom gates

Experimentally, in (S8), one can get $\zeta_x = \zeta_k = \zeta_\mu = \zeta$. It then follows that, the atom that undergoes 2D ZF and 1D SOC can be used to realize $\sqrt{-\text{PYZ}}$ gate. This is a novel gate that is a combination of $\sqrt{i\text{PY}}$ and $\sqrt{i\text{PZ}}$ gates. The solution of the governing equation when $\theta_2 = 2\pi$, in the subspace, spanned by $\{|\uparrow\rangle, |\downarrow\rangle\}$, is described by the following dynamical evolution:

$$\begin{aligned}\hat{U}_{p,v}(t) = e^{\frac{-\gamma t}{2\hbar}}\{&[\cos(\frac{\Gamma_a t}{2\hbar}) - \frac{(\gamma - i\zeta)}{\Gamma_a}\sin(\frac{\Gamma_a t}{2\hbar})]|\uparrow\rangle\langle\uparrow| + \frac{\zeta(1-i)}{\Gamma_a}\sin(\frac{\Gamma_a t}{2\hbar})|\uparrow\rangle\langle\downarrow| \\ &- \frac{\zeta(1+i)}{\Gamma_a}\sin(\frac{\Gamma_a t}{2\hbar})|\downarrow\rangle\langle\uparrow| + [\cos(\frac{\Gamma_a t}{2\hbar}) + \frac{(\gamma - i\zeta)}{\Gamma_a}\sin(\frac{\Gamma_a t}{2\hbar})]|\downarrow\rangle\langle\downarrow|\},\end{aligned} \quad \text{(S9)}$$

where $\Gamma_a = \sqrt{2\zeta^2 + (\zeta + i\gamma)^2}$. On applying a $\frac{\pi}{3}$-pulse ($2\pi\hbar = 3\sqrt{3}\zeta t$), the evolution $\hat{U}_{p,v}(\frac{2\pi\hbar}{3\sqrt{3}\zeta})$ can provide the dissipative $\sqrt{-\text{PYZ}}$ gate, as in the following:

$$\begin{aligned}\sqrt{-\text{PYZ}} = e^{\frac{-\pi\gamma}{\sqrt{27}\zeta}}\{&[\cos(\Gamma_p) - \frac{(\frac{\gamma}{\zeta} - i)}{\Gamma_1}\sin(\Gamma_p)]|\uparrow\rangle\langle\uparrow| + \frac{(1-i)}{\Gamma_1}\sin(\Gamma_p)|\uparrow\rangle\langle\downarrow| \\ &- \frac{(1+i)}{\Gamma_1}\sin(\Gamma_p)|\downarrow\rangle\langle\uparrow| + [\cos(\Gamma_p) + \frac{(\frac{\gamma}{\zeta} - i)}{\Gamma_1}\sin(\Gamma_p)]|\downarrow\rangle\langle\downarrow|\},\end{aligned} \quad \text{(S10)}$$

with $\zeta\Gamma_1 = \Gamma_a$ and $\Gamma_p = \frac{\pi\Gamma_1}{\sqrt{27}}$. The Raman beams can be applied to any individual atom in the crystal to generate 1D ZF along x-axis and 1D SOC along z-axis. Thus, the Hamiltonian (S8) with $\varpi_p \to 0$ and $\theta_2 = \pi$, can be expressed as:

$$\hat{H}_I = \zeta_x\hat{S}_v^x - \zeta_\mu\hat{S}_v^z - i\gamma\hat{S}_v^+\hat{S}_v^-, \quad \text{(S11)}$$

The temporal evolution of the system (S11) with proper timing can be used to realize the new one-atom gate $\sqrt{-\mathrm{I}_2}$ (called the square root of the minus identity operator). Particularly, when the configuration is designed such that $\zeta_x = \zeta_\mu = \zeta$, the solution of Eq.(S11) is given by:

$$\begin{aligned}\hat{U}_{I,v}(t) &= e^{\frac{-\gamma t}{2\hbar}}\{[\cos(\frac{\Gamma_b t}{2\hbar}) - \frac{(\gamma - i\zeta)}{\Gamma_b}\sin(\frac{\Gamma_b t}{2\hbar})]|\uparrow\rangle\langle\uparrow| - \frac{i\zeta}{\Gamma_b}\sin(\frac{\Gamma_b t}{2\hbar})[|\uparrow\rangle\langle\downarrow| \\ &\quad +|\downarrow\rangle\langle\uparrow|] + [\cos(\frac{\Gamma_b t}{2\hbar}) + \frac{(\gamma - i\zeta)}{\Gamma_b}\sin(\frac{\Gamma_b t}{2\hbar})]|\downarrow\rangle\langle\downarrow|\}, \end{aligned} \tag{S12}$$

with $\Gamma_b = \sqrt{\zeta^2 + (\zeta + i\gamma)^2}$. Then, on applying a $\frac{\pi}{2}$-pulse ($\pi\hbar = \sqrt{2}\zeta t$), the operation $\hat{U}_{I,v}(\frac{\pi\hbar}{\sqrt{2}\zeta})$ leads to the damped $\sqrt{-\mathrm{I}_2}$ gate, as in the following:

$$\begin{aligned}\sqrt{-\mathrm{I}_2} &= e^{\frac{-\pi\gamma}{\sqrt{8}\zeta}}\{[\cos(\Gamma_I) - \frac{(\frac{\gamma}{\zeta} - i)}{\Gamma_2}\sin(\Gamma_I)]|\uparrow\rangle\langle\uparrow| - \frac{i}{\Gamma_2}\sin(\Gamma_I)[|\uparrow\rangle\langle\downarrow| \\ &\quad +|\downarrow\rangle\langle\uparrow|] + [\cos(\Gamma_I) + \frac{(\frac{\gamma}{\zeta} - i)}{\Gamma_2}\sin(\Gamma_I)]|\downarrow\rangle\langle\downarrow|\}, \end{aligned} \tag{S13}$$

where $\zeta\Gamma_2 = \Gamma_b$ and $\Gamma_I = \frac{\pi\Gamma_2}{\sqrt{8}}$. For a non-defect system or for vanishing 1D SOC, such that $\gamma = 0$, one can obtain the ideal gates of $\sqrt{-\mathrm{PYZ}}$ and $\sqrt{-\mathrm{I}_2}$. Hence, such ideal gates in the computational space $\{|\uparrow\rangle, |\downarrow\rangle\}$, are given by:

$$\sqrt{-\mathrm{PYZ}} = \frac{1}{2}\begin{pmatrix} (1+i) & (1-i) \\ -(1+i) & (1-i) \end{pmatrix} \quad \text{and} \quad \sqrt{-\mathrm{I}_2} = \frac{1}{\sqrt{2}}\begin{pmatrix} i & -i \\ -i & -i \end{pmatrix}. \tag{S14}$$

where $|\uparrow\rangle \equiv |1\rangle = \begin{pmatrix} 1 \\ 0 \end{pmatrix}$ and $|\downarrow\rangle \equiv |0\rangle = \begin{pmatrix} 0 \\ 1 \end{pmatrix}$. Furthermore, for $\frac{3\pi}{2}$-pulse, we obtain $\hat{U}_{I,v}(\frac{3\pi\hbar}{\sqrt{2}\zeta}) \Rightarrow \sqrt{-\mathrm{I}_2}^\dagger$. The new gates (S10,S13) will be later employed to implement new circuits for controlled not (CN) gate and controlled controlled not (CCN) gate.

A.2. Other one-atom gates

For coexisting atoms at a site with Raman lasers applied to individual atoms, the atoms can produce 1D SOC around z-axis with 1D ZF around any direction. Hence, one can realize extra dissipative one-atom gates for proper time pulses during the evolution of the atom. Specifically, one can generate 1D SOC and 1D ZF along x-direction when $\zeta_k, \zeta_\mu, \vee_z \to 0$ and $\zeta_x = -\zeta$, and also 1D SOC and 1D ZF along y-direction when $\zeta_x, \zeta_\mu, \vee_z \to 0$ and $\zeta_k = \zeta$. Thus, the time evolution of (S8) for the two cases is given by:

$$\begin{aligned}\hat{U}_{x(y),v}(t) &= e^{-\frac{\gamma t}{2\hbar}}\{[\cos(\frac{\Gamma_3 t}{2\hbar}) - \frac{\gamma}{\Gamma_3}\sin(\frac{\Gamma_3 t}{2\hbar})]|\uparrow\rangle\langle\uparrow| + \frac{i(1)\zeta}{\Gamma_3}\sin(\frac{\Gamma_3 t}{2\hbar})|\uparrow\rangle\langle\downarrow| \\ &\quad + \frac{i(-1)\zeta}{\Gamma_3}\sin(\frac{\Gamma_3 t}{2\hbar})|\downarrow\rangle\langle\uparrow| + [\cos(\frac{\Gamma_3 t}{2\hbar}) + \frac{\gamma}{\Gamma_3}\sin(\frac{\Gamma_3 t}{2\hbar})]|\downarrow\rangle\langle\downarrow|\}, \end{aligned} \tag{S15}$$

where $\Gamma_3 = \sqrt{\zeta^2 - \gamma^2}$. Also, in the cases of $\zeta_x, \zeta_k, \vee_z \to 0$ and $\zeta_\mu = \zeta$, the system (S8) reads:

$$\hat{H}_z = -\zeta\hat{S}^z_v - i\gamma\hat{S}^+_v\hat{S}^-_v. \tag{S16}$$

The temporal evolution of system (S16) is given by:

$$\hat{U}_{z,v}(t) = \exp(\frac{i\zeta t - 2\gamma t}{2\hbar})|\uparrow\rangle\langle\uparrow| + \exp(-\frac{i\zeta t}{2\hbar})|\downarrow\rangle\langle\downarrow|. \tag{S17}$$

We observe that after performing specific pulses through the evolution (S15,S17), the dissipative gates of the imaginary Pauli operators and their square roots can be accomplished as in the following:

$$\hat{U}_{z,v}(\frac{\pi\hbar}{2\zeta}) = e^{i\frac{\pi}{4}}e^{\frac{-\pi\gamma}{2\zeta}}|\uparrow\rangle\langle\uparrow| + e^{-i\frac{\pi}{4}}|\downarrow\rangle\langle\downarrow| \to \sqrt{i\mathrm{PZ}}. \tag{S18}$$

$$-i\hat{U}_{z,v}(\frac{\pi\hbar}{\zeta}) = e^{\frac{-\pi\gamma}{\zeta}}|\uparrow\rangle\langle\uparrow| - |\downarrow\rangle\langle\downarrow| \rightarrow \text{PZ}. \tag{S19}$$

$$\hat{U}_{y,v}(\frac{\pi\hbar}{\zeta}) = e^{-\frac{\pi\gamma}{2\zeta}}\{[\cos(\frac{\pi\Gamma_3}{2\zeta}) - \frac{\gamma}{\Gamma_3}\sin(\frac{\pi\Gamma_3}{2\zeta})]|\uparrow\rangle\langle\uparrow| + \frac{\zeta}{\Gamma_3}\sin(\frac{\pi\Gamma_3}{2\zeta})|\uparrow\rangle\langle\downarrow| - \frac{\zeta}{\Gamma_3}\sin(\frac{\pi\Gamma_3}{2\zeta})|\downarrow\rangle\langle\uparrow| + [\cos(\frac{\pi\Gamma_3}{2\zeta}) + \frac{\gamma}{\Gamma_3}\sin(\frac{\pi\Gamma_3}{2\zeta})]|\downarrow\rangle\langle\downarrow|\} \rightarrow i\text{PY}. \tag{S20}$$

In previous studies, one-atom gates were employed using auxiliary gates to build some of the various circuits for CN-gate. These circuits can be accomplished through the evolution (S15,S17), for $\gamma = 0$, as in the following:

$$\hat{U}_{r_1,v}(\frac{\pi\hbar}{2\zeta}) \Rightarrow R_{r_1}(\frac{-\pi}{2});\ \hat{U}_{r_1,v}(\frac{7\pi\hbar}{2\zeta}) \Rightarrow R_{r_1}(\frac{\pi}{2});\ \hat{U}_{z,v}(\frac{3\pi\hbar}{\zeta}) \Rightarrow R_z(\pi),\ \text{and}\ r_1 = x, y, z. \tag{S21}$$

Also, for $\gamma = 0$, we have:

$$i\hat{U}_{y,v}(\frac{\pi\hbar}{2\zeta})\hat{U}_{z,v}(\frac{\pi\hbar}{\zeta}) \Rightarrow \text{Hadamard}(\mathcal{H}) - \text{gate}. \tag{S22}$$

This means that the cost of $\mathcal{H}$-gate is two gates. The phase-rotation gate can be realized by the specific angle $\text{Ph}(\vartheta_R) = \exp(i\vartheta_R/2)\text{I}_2$, which is employed as an auxiliary gate for the CN circuits. Moreover, by taking into account other rotation gates, such as $R_{\mathbf{n}_1}(\frac{\pi}{3})$ and $R_{\mathbf{n}_2}(\frac{\pi}{3})$ about specific axes $\mathbf{n}_1 = \frac{1}{\sqrt{3}}(1, 1, -1)$ and $\mathbf{n}_2 = \frac{1}{\sqrt{3}}(-1, 1, 1)$, the total time to accomplish these gates can be estimated to be τ_1.

B. Idealistic two-atom gates

The purpose of this section is to realize new two-atom gates (including gates with no dissipation). On tuning the Raman beams to be freely projected anywhere in the lattice during the realization of SOC and ZF, it possible to achieve interacting system with no defects. Consequently, the imaginary part of the proposed Hamiltonian vanishes. On adjusting the strengths of the tunneling, repulsion, ZF and SOC effects so that J_z is identical to $5J_y$, and D_x and D_y are eliminated, the effective model (S6) for $\theta_1 = \pi$ can be given by :

$$\hat{H}_{ef} = \frac{1}{2}\sum_{v,j=1}^{\wp}[\sqrt{J_y^2 + D_z^2}(e^{i\varphi}\hat{S}_v^+\hat{S}_j^- + e^{-i\varphi}\hat{S}_v^-\hat{S}_j^+) + 10J_y\hat{S}_v^z\hat{S}_j^z] - \sum_{v=1}^{\wp} f_z\hat{S}_v^z + \aleph\hat{I}, \tag{S23}$$

where $\varphi = \arctan(\frac{D_z}{J_y})$. The above rotation in (S23) produces an antiferromagnetic or ferromagnetic Heisenberg chain, and it gauges away the DMI effect. For the case of $\frac{D_z}{J_y} \gtrsim 1$, the spiral magnetic fields rapidly rotate, with minimum at $\frac{D_z}{J_y} \simeq 0$. The last term of Eq.(S23) has no effect on the gates realizations and, thus, can be eliminated by using the time-dependent transformation $\exp(i\aleph\hat{I}t)$. Using the canonical transformation $\hat{\digamma}_{vj} = \exp[\frac{i\varphi}{2}(\hat{S}_j^z - \hat{S}_v^z)]$, the Hamiltonian (S23) can be rewritten as in the following:

$$\hat{H}_{eff} = \hat{\digamma}_{vj}\hat{H}_{ef}\hat{\digamma}_{vj}^{\dagger} = \frac{1}{2}\sum_{v,j=1}^{\wp}\{\sqrt{J_y^2 + D_z^2}(\hat{S}_v^+\hat{S}_j^- + \hat{S}_v^-\hat{S}_j^+) + 10J_y\hat{S}_v^z\hat{S}_j^z\} - \sum_{v=1}^{\wp} f_z\hat{S}_v^z. \tag{S24}$$

The DMI can be weaker than the exchange couplings as estimated in [11]. By properly manipulating the experimental-setup of the system, we can tune the parameters of SOC and ZF to be larger or smaller than the tunneling parameters.Different solid-state experiments [12] suggest that the value of $\frac{D_z}{J_y}$ is restricted to the interval $[-5, 5]$. Here, for our ultracold atomic gas scheme, we consider $\frac{D_z}{J_y}$ in this interval. The constraints of $D_z \gtrless J_y$ help us to realize some of the quantum gates. The atoms interaction that is governed by the system (S24) include atoms at the same site, atoms at neighboring sites, pair atoms with nearest neighboring, and pair atoms with next-to-nearest neighboring (i.e., between atom pairs 1-2, 2-3, 1-3 and so on). We focus our attention to only two atoms pairs. The time evolution of the system (S24) for two interacting atoms across site 1 and 2 ($\wp = 2$) can be expressed as in the following:

$$\hat{U}(t) = e^{-i\varphi_2 t}|\uparrow\uparrow\rangle\langle\uparrow\uparrow| + e^{-i\theta t}\{\cos(\theta t)(|\uparrow\downarrow\rangle\langle\uparrow\downarrow| + |\downarrow\uparrow\rangle\langle\downarrow\uparrow|) - i\sin(\theta t)(|\uparrow\downarrow\rangle\langle\downarrow\uparrow| + |\downarrow\uparrow\rangle\langle\uparrow\downarrow|)\} + e^{-i\varphi_1 t}|\downarrow\downarrow\rangle\langle\downarrow\downarrow|, \tag{S25}$$

where $\theta = \frac{\sqrt{J_y^2+D_z^2}}{\hbar}$, $\varphi_1 = \theta + \frac{(5J_y+f_z)}{\hbar}$ and $\varphi_2 = \theta + \frac{(5J_y-f_z)}{\hbar}$. For Zeeman parameter f_z equal J_y, the evolution (S25) becomes:

$$\hat{U}(t) = e^{-i\theta t}\begin{cases} e^{\frac{-45iJ_yt}{4\hbar}}|\uparrow\uparrow\rangle\langle\uparrow\uparrow| + \cos(\theta t)\{|\uparrow\downarrow\rangle\langle\uparrow\downarrow| + |\downarrow\uparrow\rangle\langle\downarrow\uparrow|\} \\ -i\sin(\theta t)\{|\uparrow\downarrow\rangle\langle\downarrow\uparrow| + |\downarrow\uparrow\rangle\langle\uparrow\downarrow|\} + e^{\frac{5iJ_yt}{4\hbar}}|\downarrow\downarrow\rangle\langle\downarrow\downarrow|, \\ \forall\ f_z = -\frac{25J_y}{4}. \\ e^{\frac{-25iJ_yt}{2\hbar}}|\uparrow\uparrow\rangle\langle\uparrow\uparrow| + \cos(\theta t)\{|\uparrow\downarrow\rangle\langle\uparrow\downarrow| + |\downarrow\uparrow\rangle\langle\downarrow\uparrow|\} \\ -i\sin(\theta t)\{|\uparrow\downarrow\rangle\langle\downarrow\uparrow| + |\downarrow\uparrow\rangle\langle\uparrow\downarrow|\} + e^{\frac{5iJ_yt}{2\hbar}}|\downarrow\downarrow\rangle\langle\downarrow\downarrow|, \\ \forall\ f_z = -\frac{15J_y}{2}. \end{cases} \tag{S26}$$

B.1. Proposed two-atom gates

According to [13], there are two forms of the $-$SWCZ gate: $\sqrt{-\text{SWCZ}} = \pm i\sqrt{\text{SWCZ}}$. One of them is define and generated in Ref.[14], while both will be generated in the current work. For $J_y = \frac{4\hbar\theta}{5}$ and $J_y = \frac{2\hbar\theta}{5}$, through the first and second branch of Eq.(S26), respectively, the system (S26) can be given by:

$$\hat{U}_{sz}(t) = \begin{cases} e^{-10i\theta t}|\uparrow\uparrow\rangle\langle\uparrow\uparrow| + \frac{(1+e^{-2i\theta t})}{2}\{|\uparrow\downarrow\rangle\langle\uparrow\downarrow| + |\downarrow\uparrow\rangle\langle\downarrow\uparrow|\} \\ -\frac{(1-e^{-2i\theta t})}{2}\{|\uparrow\downarrow\rangle\langle\downarrow\uparrow| + |\downarrow\uparrow\rangle\langle\uparrow\downarrow|\} + |\downarrow\downarrow\rangle\langle\downarrow\downarrow|, \\ \forall\ f_z = -\frac{25J_y}{4}. \\ e^{-6i\theta t}|\uparrow\uparrow\rangle\langle\uparrow\uparrow| + \frac{(1+e^{-2i\theta t})}{2}\{|\uparrow\downarrow\rangle\langle\uparrow\downarrow| + |\downarrow\uparrow\rangle\langle\downarrow\uparrow|\} \\ -\frac{(1-e^{-2i\theta t})}{2}\{|\uparrow\downarrow\rangle\langle\downarrow\uparrow| + |\downarrow\uparrow\rangle\langle\uparrow\downarrow|\} + |\downarrow\downarrow\rangle\langle\downarrow\downarrow|, \\ \forall\ f_z = -\frac{15J_y}{2}. \end{cases} \tag{S27}$$

After the action of a $\pi/4-$pulse ($\pi/4 = \theta t$), two new gates of the two portraits can be generated: $\hat{U}_{sz}(\frac{\pi}{4\theta}) \Rightarrow i\sqrt{\text{SWCZ}}$ gate for $f_z = -\frac{25J_y}{4}$ and $\hat{U}_{sz}(\frac{\pi}{4\theta}) \Rightarrow -i\sqrt{\text{SWCZ}}$ gate for $f_z = -\frac{15J_y}{2}$. This means that the required cost to achieve $i\sqrt{\text{SWCZ}}$ or $-i\sqrt{\text{SWCZ}}$ is one gate. In the computational space $\{|\uparrow\rangle, |\downarrow\rangle\} \otimes \{|\uparrow\rangle, |\downarrow\rangle\}$, the $\pm i\sqrt{\text{SWCZ}}$ gates can be expressed as in the following:

$$\pm i\sqrt{\text{SWCZ}} = \begin{pmatrix} \mp i & 0 & 0 & 0 \\ 0 & \frac{1}{2}(1-i) & -\frac{1}{2}(1+i) & 0 \\ 0 & -\frac{1}{2}(1+i) & \frac{1}{2}(1-i) & 0 \\ 0 & 0 & 0 & 1 \end{pmatrix}. \tag{S28}$$

B.2. Other two-atom gates

For strong DMI effect ($J_y = \frac{\hbar\theta}{4}$), the evolution is governed by (S26). By applying 2π-pulse ($2\pi = \theta t$), a controlled-z (CZ) gate can be formed, as detailed in following:

$$\begin{aligned} &\{\hat{U}_{z,1}(\tfrac{5\pi\hbar}{8\zeta}) \otimes \hat{I}_2\}\hat{U}(\tfrac{2\pi}{\theta})\{\hat{I}_2 \otimes \hat{U}_{z,2}(\tfrac{5\pi\hbar}{8\zeta})\} \Rightarrow \text{CZ}, \quad \forall\ f_z = -\tfrac{25J_y}{4}. \\ &\{\hat{U}_{z,1}(\tfrac{5\pi\hbar}{4\zeta}) \otimes \hat{I}_2\}\hat{U}(\tfrac{2\pi}{\theta})\{\hat{I}_2 \otimes \hat{U}_{z,2}(\tfrac{5\pi\hbar}{4\zeta})\} \Rightarrow \text{CZ}, \quad \forall\ f_z = -\tfrac{15J_y}{2}. \end{aligned} \tag{S29}$$

Each branch of Eq.(S29) leads to CZ-gate. This means that the cost to achieve CZ is three gates. On the other hand, to realize the square root of CZ-gate and its inverse, some non-dissipative rotation gates (of one-atom type around the z-axis with temporal evolution (S26)) are needed. Thus, if we assume that the exchange coupling is set to be equal to $\frac{3\hbar\theta}{4}$ and $\frac{\hbar\theta}{4}$ with pulse π, we can achieve these gates as in the following:

$$\begin{aligned} &\otimes_{j=1}^{2} \hat{U}_{z,j}(\tfrac{31\pi\hbar}{16\zeta})\hat{U}(\tfrac{\pi}{\theta}) \Rightarrow \sqrt{\text{CZ}} \text{ and } \otimes_{j=1}^{2} \hat{U}_{z,j}(\tfrac{21\pi\hbar}{16\zeta})\hat{U}(\tfrac{\pi}{\theta}) \Rightarrow \sqrt{\text{CZ}}^{\dagger}, \ \forall\ f_z = -\tfrac{25J_y}{4}. \\ &\otimes_{j=1}^{2} \hat{U}_{z,j}(\tfrac{23\pi\hbar}{8\zeta})\hat{U}(\tfrac{\pi}{\theta}) \Rightarrow \sqrt{\text{CZ}} \text{ and } \otimes_{j=1}^{2} \hat{U}_{z,j}(\tfrac{13\pi\hbar}{8\zeta})\hat{U}(\tfrac{\pi}{\theta}) \Rightarrow \sqrt{\text{CZ}}^{\dagger}, \ \forall\ f_z = -\tfrac{15J_y}{2}. \end{aligned} \tag{S30}$$

Furthermore, using the present system, we can implement previously implemented two-atom gates for CN-gate realization with lower cost. For instance, for weak SOC, $D_z < J_y$, and robust SOC, $D_z > J_y$, we have:

$$\begin{array}{l} e^{\frac{7i\pi}{2}}\hat{U}(\frac{7\pi}{2\theta}) \Rightarrow i\mathrm{SWAP} \;\; ; \;\; e^{\frac{7i\pi}{4}}\hat{U}(\frac{7\pi}{4\theta}) \Rightarrow \sqrt{i\mathrm{SWAP}}, \;\; \forall\; J_y = \frac{32\hbar\theta}{35} \text{ and } f_z = -\frac{25J_y}{4}. \\ e^{-\frac{i\pi}{2}}\hat{U}(\frac{3\pi}{2\theta}) \Rightarrow i\mathrm{SWAP} \;\; ; \;\; e^{-\frac{i\pi}{4}}\hat{U}(\frac{3\pi}{4\theta}) \Rightarrow \sqrt{i\mathrm{SWAP}}, \;\; \forall\; J_y = \frac{8\hbar\theta}{15} \text{ and } f_z = -\frac{15J_y}{2}. \end{array} \tag{S31}$$

Also, for $f_z = -\frac{25J_y}{4}$ and $J_y = \frac{4\hbar\theta}{5}$; $f_z = -\frac{15J_y}{2}$ and $J_y = \frac{2\hbar\theta}{5}$, we obtain

$$\hat{U}(\frac{\pi}{2\theta}) \Rightarrow -\mathrm{SWCZ}. \tag{S32}$$

By implementing the above two-atom gates, either for strong DMI or weak DMI, we can realize $\sqrt{\mathrm{SWAP}}$ gate with the evolution (S26) for the cases of $f_z = -\frac{25J_y}{4}$ and $J_y = \frac{12\hbar\theta}{25}$, or $f_z = -\frac{15J_y}{2}$ and $J_y = \frac{6\hbar\theta}{25}$, respectively. In other words, such a gate can be achieved by a series of robustness and weakness of the DMI with specific pulses during the time evolution of the system (S24). Specifically, this gate can be realized as in the following:

$$i\sqrt{\mathrm{CZ}}^{\dagger}\hat{U}(\frac{5\pi}{4\theta}) \Rightarrow \sqrt{\mathrm{SWAP}} \;\; ; \;\; i\sqrt{\mathrm{CZ}}\,\hat{U}(\frac{5\pi}{4\theta}) \Rightarrow \sqrt{\mathrm{SWAP}}. \tag{S33}$$

Similarly, when $f_z = -\frac{25J_y}{4}$ and $J_y = \frac{4\hbar\theta}{15}$, or $J_y = \frac{2\hbar\theta}{3}$ and $f_z = -\frac{15J_y}{2}$, the realization of the SWAP gate is given by:

$$-\mathrm{CZ}\,\hat{U}(\frac{3\pi}{2\theta}) \Rightarrow \mathrm{SWAP}. \tag{S34}$$

Thus, we can conceive the square root of CN ($\sqrt{\mathrm{CN}}$) gate with DMI effect as in the following:

$$\sqrt{\mathrm{CN}} \;\; = \;\; \hat{U}_{y,2}(\frac{\pi\hbar}{2\zeta})\,\sqrt{\mathrm{CZ}}\,\hat{U}_{y,2}(\frac{7\pi\hbar}{2\zeta}). \tag{S35}$$

From Eq.(S30), we can note that the cost of $\sqrt{\mathrm{CZ}}$ gate is three gates, and thus the cost of $\sqrt{\mathrm{CN}}$ gate is five gates.

C. Dissipative two-atom gates

As some defects may occur inside the lattice system due to the arising heat effect during the application of Raman lasers, dissipation takes palce. On the other hand, such defects may be arisen due to other sources (mentioned above). To take disspation into account, we obay the same transformations detailed above (S24) while the dissipation parameters are incorporated. It then follows that the dissipative Hamiltonian (S6) for any two interacting atoms (say at sites 1 and 2) can be expressed as in the following:

$$\begin{aligned} \hat{H}_{diss} \;\; &= \;\; \top_1(\hat{S}_1^+\hat{S}_2^- + \hat{S}_2^+\hat{S}_1^-) - \top_2(\hat{S}_1^+\hat{S}_1^- + \hat{S}_2^+\hat{S}_2^-) \\ &\quad -\top_3(\hat{S}_1^+\hat{S}_2^-\hat{S}_2^+\hat{S}_1^- + \hat{S}_2^+\hat{S}_1^-\hat{S}_1^+\hat{S}_2^-) + 5J_y\hat{S}_1^+\hat{S}_1^-\hat{S}_2^+\hat{S}_2^- + \top_4\hat{I}, \end{aligned} \tag{S36}$$

where $\top_1 = (\hbar\theta - 2i\gamma_2\cos\varphi)$, $\top_2 = (2\gamma_2\sin\varphi + i\gamma_1 + f_z + 2.5J_y)$, $\top_3 = (4i\gamma_2 + 2.5J_y)$ and $\top_4 = (\hbar\theta + 5J_y - 2i\gamma_2 e^{i\varphi} + f_z)$. To find the damped form, we compute the time evolution of the total unnormalized state of the wave function that is governed by the non-Hermitian Schrodinger equation, yielding:

$$\begin{aligned} \hat{U}_{diss}(t) \;\; &= \;\; e^{-i\varnothing_1 t}|\uparrow\uparrow\rangle\langle\uparrow\uparrow| + e^{-i\varnothing_2 t}\{\cos(\varnothing t)(|\uparrow\downarrow\rangle\langle\uparrow\downarrow| + |\downarrow\uparrow\rangle\langle\downarrow\uparrow|) \\ &\quad -i\sin(\varnothing t)(|\uparrow\downarrow\rangle\langle\downarrow\uparrow| + |\downarrow\uparrow\rangle\langle\uparrow\downarrow|)\} + e^{-i\varnothing_3 t}|\downarrow\downarrow\rangle\langle\downarrow\downarrow|, \end{aligned} \tag{S37}$$

where $\varnothing = \frac{\top_1}{\hbar}$, $\varnothing_1 = \theta + \frac{(5J_y - 2i\gamma_1 - f_z - 2i\gamma_2 e^{-i\varphi})}{\hbar}$, $\varnothing_2 = \varnothing - \frac{i(\gamma_1 + 4\gamma_2)}{\hbar}$ and $\varnothing_3 = \frac{\top_4}{\hbar}$. By using appropriate values: $f_z \equiv f_{\pm}J_y$, $D_z \equiv D_{\pm}J_y$, $(f_+, D_+) = (-\frac{25}{4}, \frac{3}{4})$ and $(f_-, D_-) = (-\frac{15}{2}, \frac{\sqrt{21}}{2})$, the dissipative evolution (S37) reads:

$$\begin{aligned} \hat{U}_{\pm}(t, \gamma_1, \gamma_2) \;\; &= \;\; e^{-i\varnothing_1^{\pm} t}|\uparrow\uparrow\rangle\langle\uparrow\uparrow| + e^{-i\varnothing_2^{\pm} t}\{\cos(\varnothing_{\pm} t)(|\uparrow\downarrow\rangle\langle\uparrow\downarrow| + |\downarrow\uparrow\rangle\langle\downarrow\uparrow|) \\ &\quad -i\sin(\varnothing_{\pm} t)(|\downarrow\uparrow\rangle\langle\uparrow\downarrow| + |\uparrow\downarrow\rangle\langle\downarrow\uparrow|)\} + e^{-i\varnothing_3^{\pm} t}|\downarrow\downarrow\rangle\langle\downarrow\downarrow|, \end{aligned} \tag{S38}$$

where $\varnothing_\pm = (\theta - \frac{2i\gamma_2 \cos\varphi_\pm}{\hbar})$, $\varnothing_1^\pm = (8 \pm 2)\theta - \frac{2i(\gamma_1+\gamma_2 e^{-i\varphi_\pm})}{\hbar}$, $\varnothing_2^\pm = \varnothing_\pm - \frac{i(\gamma_1+4\gamma_2)}{\hbar}$, $\varnothing_3^\pm = -\frac{2i\gamma_2 e^{i\varphi_\pm}}{\hbar}$ and $\varphi_\pm = \arctan(D_\pm)$. Analytically, we can show that $\hat{U}_\pm(\frac{\pi}{4\theta}, \gamma_1, \gamma_2)$ can be used to from dissipative $\pm i\sqrt{\text{SWCZ}}$ gates. On the other hand, on having $D^+ = \frac{\sqrt{7}J_y}{3}$, $D^- = \sqrt{15}J_y$ and $f_z = f_+ J_y$ for all $D^\pm$, the evolution (S37) yields:

$$\begin{aligned}\hat{U}^\pm(\frac{\pi}{\theta}, \gamma_1, \gamma_2) \quad &= \quad e^{-i\S_1^\pm} |\uparrow\uparrow\rangle\langle\uparrow\uparrow| + e^{-i\S_2^\pm}\{\cos(\S_\pm)(|\uparrow\downarrow\rangle\langle\uparrow\downarrow| + |\downarrow\uparrow\rangle\langle\downarrow\uparrow|) \\ &\quad -i\sin(\S_\pm)(|\downarrow\uparrow\rangle\langle\uparrow\downarrow| + |\uparrow\downarrow\rangle\langle\downarrow\uparrow|)\} + e^{-i\S_3^\pm} |\downarrow\downarrow\rangle\langle\downarrow\downarrow|, \end{aligned} \tag{S39}$$

where $\S_\pm = (\pi - \frac{2i\pi\gamma_2 \cos\vartheta_\pm}{\hbar\theta})$, $\vartheta_\pm = \arctan(D^\pm)$, $\S_1^\pm = \frac{(106\pm45)\pi}{16} - \frac{2i\pi(\gamma_1+\gamma_2 e^{-i\vartheta_\pm})}{\hbar\theta}$, $\S_2^\pm = \S_\pm - \frac{i\pi(\gamma_1+4\gamma_2)}{\hbar\theta}$ and $\S_3^\pm = \frac{(6\mp5)\pi}{16} - \frac{2i\pi\gamma_2 e^{i\vartheta_\pm}}{\hbar\theta}$. By functioning the rotation gates of Eq.(S17) [which are $\hat{U}_{z,v}(\frac{31\pi\hbar}{16\zeta})$, $\hat{U}^+$, $\hat{U}_{z,v}(\frac{21\pi\hbar}{16\zeta})$ and $\hat{U}^-$] we can realize the damped $\sqrt{\text{CZ}}$ gate and its inverse.

D. Constraints to realize quantum gates

The chosen constraints to realize the proposed gates with SOC and ZF mechanisms are reached under several considerations. These include: (i) for other constraints than the above, say $f_z = \pm \sqcup_1 J_y$ and $J_z = \sqcup_2 J_y$ $(0 < \sqcup_1 < 1, \sqcup_2 < 5)$, the realization for each two-atom gate requires larger number of gates. (ii) for other constraints of J_y, say $J_y = \sqcup_3 \hbar\theta$ with $\sqcup_3 > 1$, the ratio $\frac{D_z}{J_y}$ will be with imaginary value. Also, for J_y with $\sqcup_3 < 1$, larger number of gates are required and the DMI effect becomes an extremely strong. (iii) the gate $\pm i\sqrt{\text{SWCZ}}$ can not be realized by taking other values for J_y. On the other hand, previous studies of the optical lattice without SOC and ZF mechanisms (which are governed by $\hat{H}_\pm = \sum_{v,j=1}^{2}(\pm J_1 \hat{\sigma}_v^x \hat{\sigma}_j^x \pm J_1 \hat{\sigma}_v^y \hat{\sigma}_j^y + J_2 \hat{\sigma}_v^z \hat{\sigma}_j^z) - \sum_v f^z \hat{\sigma}_v^z$) require the value of J_2 to be larger than or equal the coupling J_1 to achieve the XXZ gate. Therefore, the time evolution of $\hat{H}_\pm$ requires a series of gates to realize CZ-gate. For instance, this evolution needs at least one auxiliary gate of the two-atom kind (controlled-phase gate) and two auxiliary gates of the one-atom kind for two-atom gates of Eqs.(S30,S31,S33,S34). Moreover, the evolution of $\hat{H}_\pm$ requires two gates of the one-atom type to achieve $\pm i\sqrt{\text{SWCZ}}$ gates. Consequently, our two-atom gates require lower cost than the two-atom gates in the absence of the DMI or SOC effect. Hereby, it is shown that our realization of Eqs.(S28-S34) is more efficient than their previous realization.

3. DETAILS ON THE ALGORITHM TO SOLVE SP

Overview: We know that the idealistic Simon problem (SP) is a polynomial-time algorithm and it can distinguish between two complexity classes by employing a polynomial-time function. The first-class is the bounded error probabilistic polynomial-time, which can be solved by a Turing machine. The second-class is the bounded-error quantum polynomial-time, which may be solved by a quantum computer with error probability less than first class. Besides, if the function of the Turing machine is used as an oracle or circuit in quantum, then it requires an exponential number of oracle evaluations to solve the problem. Thus, the solution to the problem is difficult for the classic conception while it will be exponentially faster with the quantum conception.

A. Design of novel oracles

Each possible circuits (48 circuits), which represent the dissipative oracles $B_{\mathfrak{F}}(\gamma_1, \gamma_2)$ to evaluate the function $\mathfrak{F}$ of the two-atom algorithm, given in Fig.(S3). These circuits are produced using the configuration of CN, I_2 and iPY gates, which own the state $|\phi_2\rangle = -\frac{1}{2}\{|00\rangle_1 + |01\rangle_1 + |10\rangle_1 + |11\rangle_1\} \otimes |00\rangle_2$, inside $\wp_1$, $\wp_2$ sites. Consequently, after performing the three first steps of the algorithm to generate 48 various schemes to solve a problem, we find that each one of these schemes elucidates and decides that the function $\mathfrak{F}$ is a $4 - \text{to} - 1$ type function. For simple clarification, we present in the table (S1) the outputs of $\wp_2$-site to determine $\mathfrak{F}$ for each scheme in the ideal case. In such table, the outputs of sch_1-sch_{36} show that $\mathfrak{F}$ is an $2 - \text{to} - 1$ function, and the outputs of sch_{37}-sch_{48} elucidate that $\mathfrak{F}$ is an $1 - \text{to} - 1$ function. We present in Figs.(S4,S5,S6) new forty-seven oracles of $B_{\mathfrak{F}}(\gamma_1, \gamma_2)$ to determine and compute the function $\mathfrak{F}$ of the three-atom algorithm. Such oracles are generating during the configuration of CCN, CN, I_2 and iPY gates across the atoms that coexist in $\wp_1$, $\wp_2$ sites and occupy the state $|\phi_2\rangle = \frac{i}{2\sqrt{2}}\{|000\rangle_1 + |001\rangle_1 + |010\rangle_1 + |011\rangle_1 + |100\rangle_1 + |101\rangle_1 + |110\rangle_1 + |111\rangle_1\} \otimes |000\rangle_2$. As above,

Outputs	sch_1	sch_2	sch_3	sch_4	sch_5	sch_6	sch_7	sch_8	sch_9	sch_{10}	sch_{11}	sch_{12}	sch_{13}	sch_{14}	sch_{15}
$\mathfrak{F}(00)$	00	00	00	01	01	01	10	10	10	11	11	11	00	00	00
$\mathfrak{F}(01)$	01	10	11	00	10	11	00	01	11	00	01	10	01	10	11
$\mathfrak{F}(10)$	01	10	11	00	10	11	00	01	11	00	01	10	00	00	00
$\mathfrak{F}(11)$	00	00	00	01	01	01	10	10	10	11	11	11	01	10	11
No. Circles	(a_3)	(a_2)	(a_1)	(a_3)	(a_1)	(a_2)	(a_2)	(a_1)	(a_3)	(a_1)	(a_2)	(a_3)	(a_6)	(a_5)	(a_4)
	$\mathcal{G}_1, \mathcal{G}_2 = I_2$			$\mathcal{G}_1 = I_2, \mathcal{G}_2 = i$PY			$\mathcal{G}_1 = i$PY$, \mathcal{G}_2 = I_2$			$\mathcal{G}_1, \mathcal{G}_2 = i$PY			$\mathcal{G}_1, \mathcal{G}_2 = I_2$		

sch_{16}	sch_{17}	sch_{18}	sch_{19}	sch_{20}	sch_{21}	sch_{22}	sch_{23}	sch_{24}	sch_{25}	sch_{26}	sch_{27}	sch_{28}	sch_{29}	sch_{30}	sch_{31}	sch_{32}	sch_{33}
01	01	01	10	10	10	11	11	11	00	00	00	01	01	01	10	10	10
00	10	11	00	01	11	00	01	10	00	00	00	01	01	01	10	10	10
01	01	01	10	10	10	11	11	11	01	10	11	00	10	11	00	01	11
00	10	11	00	01	11	00	01	10	01	10	11	00	10	11	00	01	11
(a_6)	(a_4)	(a_5)	(a_5)	(a_4)	(a_6)	(a_4)	(a_5)	(a_6)	(a_9)	(a_8)	(a_7)	(a_9)	(a_7)	(a_8)	(a_8)	(a_7)	(a_9)
$\mathcal{G}_1 = I_2, \mathcal{G}_2 = i$PY			$\mathcal{G}_1 = i$PY$, \mathcal{G}_2 = I_2$			$\mathcal{G}_1, \mathcal{G}_2 = i$PY			$\mathcal{G}_1, \mathcal{G}_2 = I_2$			$\mathcal{G}_1 = I_2, \mathcal{G}_2 = i$PY			$\mathcal{G}_1 = i$PY$,\mathcal{G}_2 = I_2$		

sch_{34}	sch_{35}	sch_{36}	sch_{37}	sch_{38}	sch_{39}	sch_{40}	sch_{41}	sch_{42}	sch_{43}	sch_{44}	sch_{45}	sch_{46}	sch_{47}	sch_{48}
11	11	11	00	00	00	01	01	01	10	10	10	11	11	11
11	11	11	01	10	11	10	11	00	01	11	00	00	01	10
00	01	10	10	11	01	11	00	10	11	00	01	01	10	00
00	01	10	11	01	10	00	10	11	00	01	11	10	00	01
(a_7)	(a_8)	(a_9)	(a_{10})	(a_{11})	(a_{12})	(a_{13})	(a_{14})	(a_{15})	(a_{12})	(a_{10})	(a_{11})	(a_{13})	(a_{14})	(a_{15})
$\mathcal{G}_1, \mathcal{G}_2 = i$PY			$\mathcal{G}_1 = I_2$			$\mathcal{G}_1 = I_2, \mathcal{G}_2 = i$PY			$\mathcal{G}_1 = i$PY			$\mathcal{G}_1, \mathcal{G}_2 = i$PY		

Table S1. The outputs of $\wp_2$-site after finishing the three first experimental requirements for each scheme of the two-atom in the absence of dissipation.

one can find that each one of these 47 schemes demonstrates that $\mathfrak{F}$ is an $8-$ to -1 type function. Also, we show in the table (S2) the algorithm outputs of the three first steps for each second register of the schemes without dissipation. Thus, we find from the table (S2) that the schemes outputs of b_1-b_{35} decide that $\mathfrak{F}$ is an $2-$ to -1 function, and the schemes of b_{36}-b_{47} decide that $\mathfrak{F}$ is an $1-$ to -1 function. Furthermore, we demonstrate that the remaining oracles of $B_{\mathfrak{F}}$ are often similar to some of these oracles but with performing iPY and I_2 gates or no upon any state of second register $|000\rangle_2$.

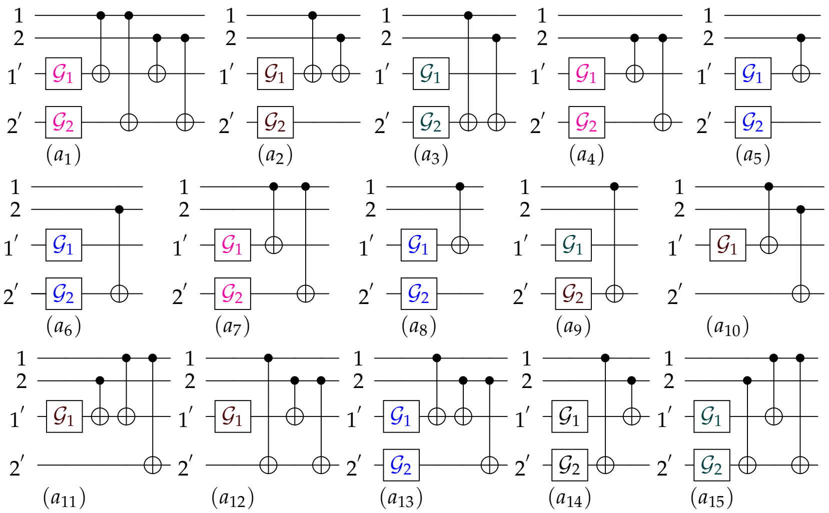

Fig. S3. The possible new oracles to evaluate $\mathfrak{F}$ with the dissipation to obtain the solution of SP of the two-atom algorithm. The top indexes 1,2 refer to the atoms of $\wp_1$-site while the bottom indexes $1', 2'$ referring to the atoms of $\wp_2$-site. The gates $(\mathcal{G}_1, \mathcal{G}_2)$ for each circuit are defined in the table (S1).

Outputs	(b_1)	(b_2)	(b_3)	(b_4)	(b_5)	(b_6)	(b_7)	(b_8)	(b_9)	(b_{10})	(b_{11})	(b_{12})	(b_{13})	(b_{14})	(b_{15})
$\mathfrak{F}(000)$	000	000	000	000	000	001	001	001	001	001	010	010	010	010	010
$\mathfrak{F}(001)$	000	000	000	000	000	010	011	100	101	110	011	100	101	110	111
$\mathfrak{F}(010)$	001	010	011	100	101	001	001	001	001	001	011	100	101	110	111
$\mathfrak{F}(011)$	001	010	011	100	101	010	011	100	101	110	010	010	010	010	010
$\mathfrak{F}(100)$	010	011	100	101	110	011	100	101	110	111	100	101	110	111	000
$\mathfrak{F}(101)$	010	011	100	101	110	100	101	110	111	000	101	110	111	000	001
$\mathfrak{F}(110)$	011	100	101	110	111	011	100	101	110	111	101	110	111	000	001
$\mathfrak{F}(111)$	011	100	101	110	111	100	101	110	111	000	100	101	110	111	000

(b_{16})	(b_{17})	(b_{18})	(b_{19})	(b_{20})	(b_{21})	(b_{22})	(b_{23})	(b_{24})	(b_{25})	(b_{26})	(b_{27})	(b_{28})	(b_{29})	(b_{30})
011	011	011	011	011	100	100	100	100	100	101	101	101	101	101
100	101	110	111	000	101	110	111	000	001	110	111	000	010	001
101	110	111	000	001	110	111	000	001	010	111	000	001	011	010
110	111	000	001	010	111	000	001	010	011	000	001	010	100	011
011	011	011	011	011	101	110	111	000	001	111	000	001	011	010
100	101	110	111	000	100	100	100	100	100	000	001	010	100	011
101	110	111	000	001	111	000	001	010	011	101	101	101	101	101
110	111	000	001	010	110	111	000	001	010	110	111	000	010	001

(b_{31})	(b_{32})	(b_{33})	(b_{34})	(b_{35})	(b_{36})	(b_{37})	(b_{38})	(b_{39})	(b_{40})	(b_{41}	(b_{42})	(b_{43})	(b_{44})	(b_{45})	(b_{46})	(b_{47})
110	110	110	111	111	000	001	010	011	000	001	010	110	100	101	111	111
000	001	010	011	100	001	010	011	100	110	101	000	101	111	100	110	011
001	010	011	100	110	010	011	100	101	111	110	001	111	000	110	000	100
010	011	100	101	000	011	100	101	110	001	111	011	000	001	111	001	101
010	011	100	101	000	100	101	110	111	010	000	100	001	010	000	010	110
001	010	011	100	110	101	110	111	000	011	010	101	010	011	001	011	000
000	001	010	011	100	110	111	000	001	100	011	110	011	101	010	100	001
110	110	110	111	111	111	000	001	010	101	100	111	100	110	011	101	010

Table S2. The outputs of $\wp_2$-site to evaluate $\mathfrak{F}$ without dissipation for each scheme of the three-atom algorithm.

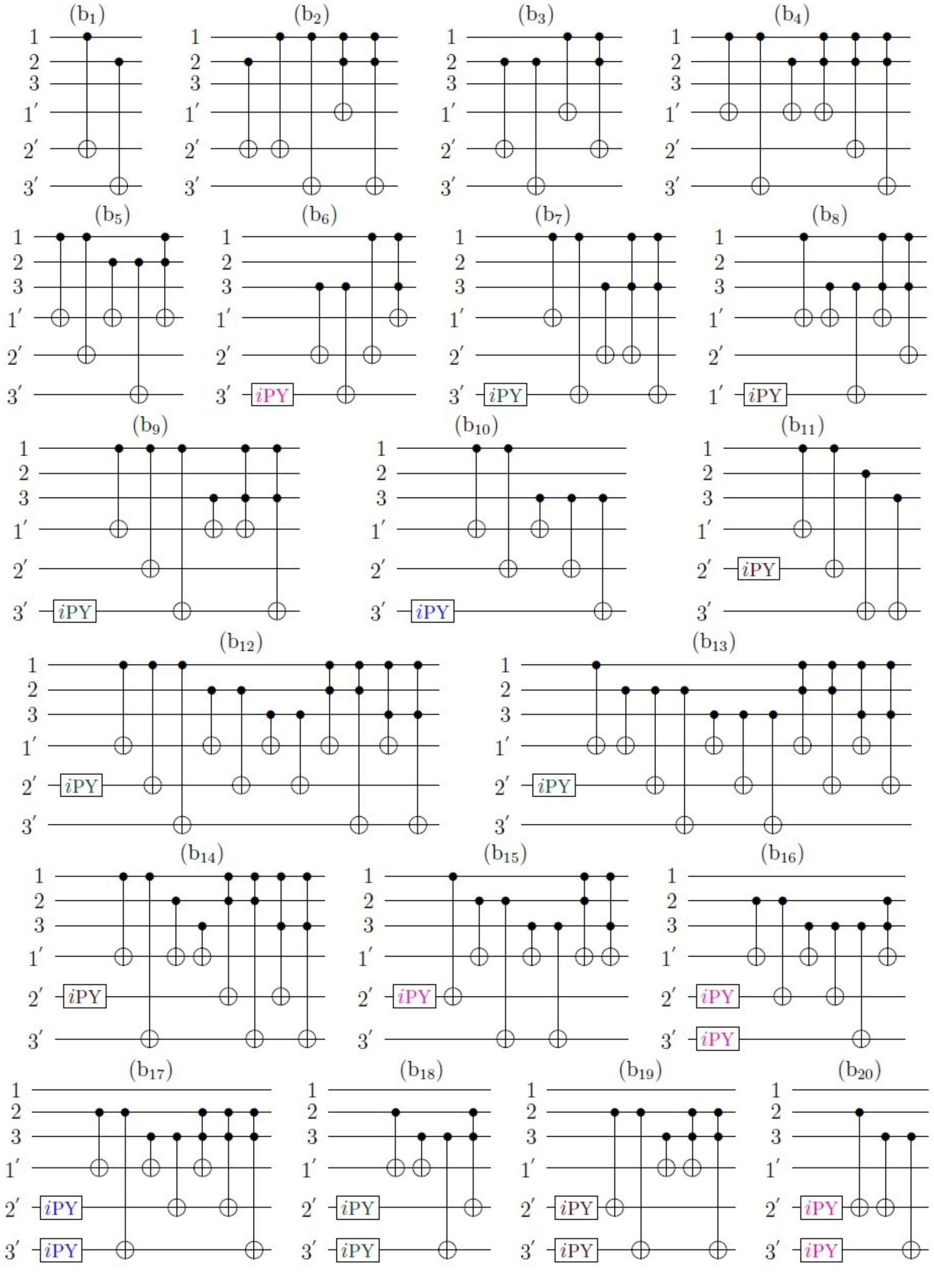

Fig. S4. New oracles of $B_{\mathfrak{F}}(\gamma_1, \gamma_2)$ to determine $\mathfrak{F}$ for solving SP of a 3-atom algorithm.

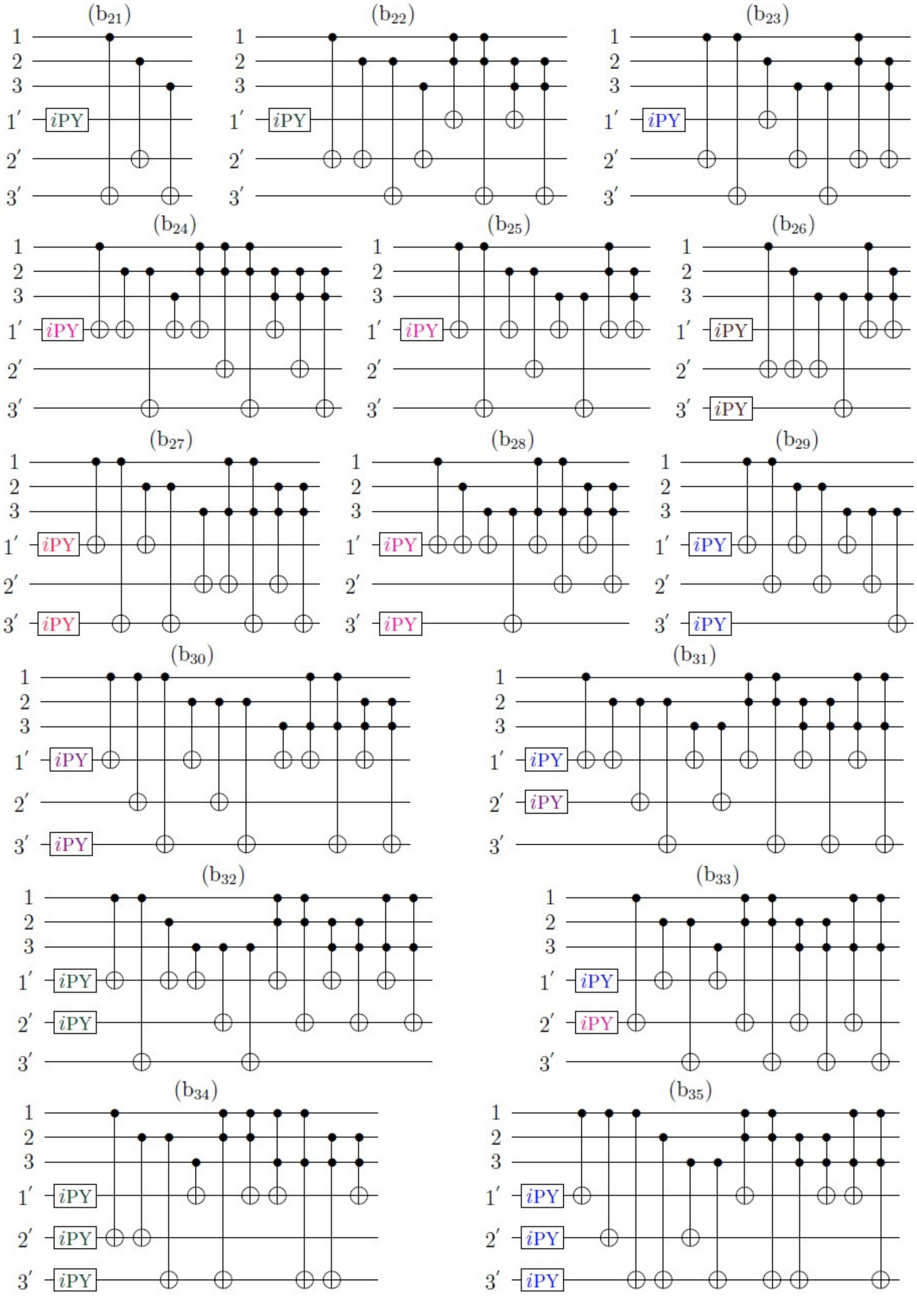

Fig. S5. Other new $B_{\mathfrak{F}}(\gamma_1, \gamma_2)$ as Fig.(S4) to evaluate $\mathfrak{F}$.

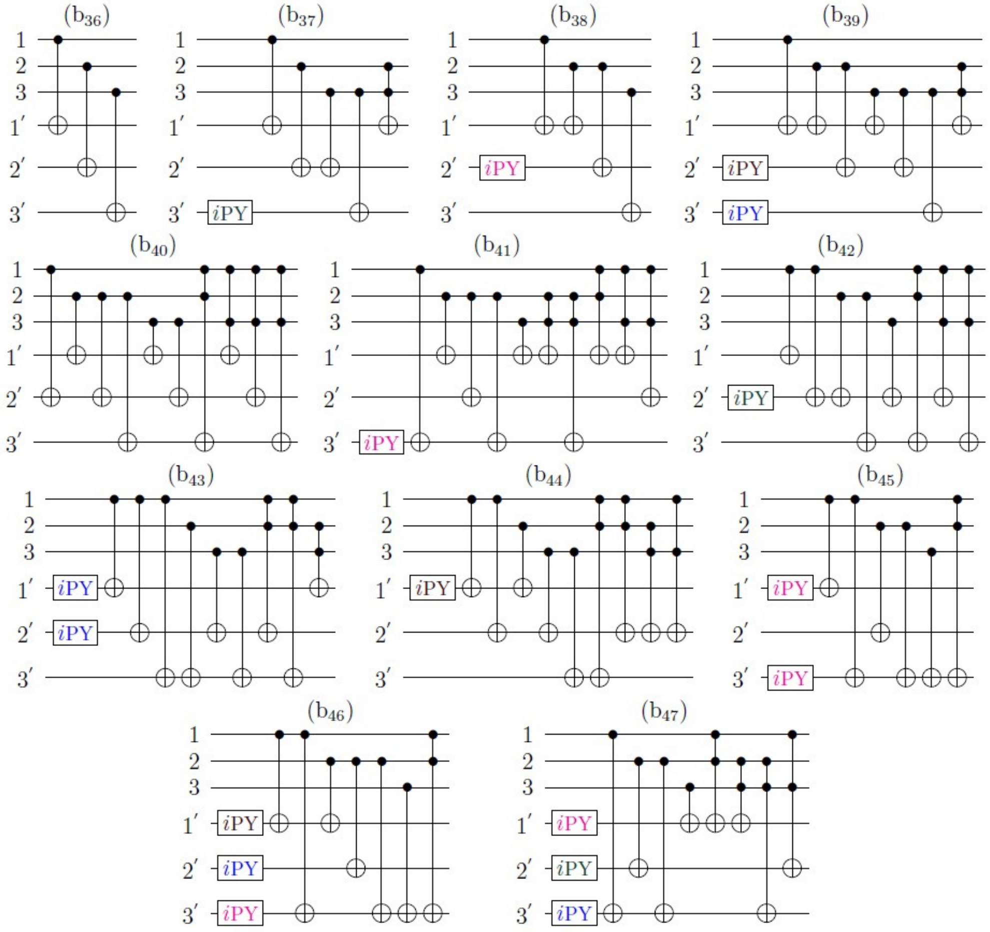

Fig. S6. Other circuits of $B_{\mathfrak{F}}(\gamma_1, \gamma_2)$ that describe the remaining novel oracles as Figs.(S4,S5).

B. Example to explain the string selection

- The two registers' initial state is prepared as: $|\phi_1\rangle = |000\rangle_1|000\rangle_2$.
- Perform the gate, $\sqrt{-\mathrm{I}_2} = \frac{i}{\sqrt{2}}\{|1\rangle\langle 1| - |1\rangle\langle 0| - |0\rangle\langle 1| - |0\rangle\langle 0|\}$, to each atom of the first register, we have $|\phi_1\rangle \to |\phi_2\rangle = \frac{i}{2\sqrt{2}}\{|000\rangle_1 + |001\rangle_1 + |010\rangle_1 + |011\rangle_1 + |100\rangle_1 + |101\rangle_1 + |110\rangle_1 + |111\rangle_1\}|000\rangle_2$.
- Perform the ideal oracle of Fig.(S4b$_{11}$) to occupied atoms of $\wp_1$, $\wp_2$ sites, so $|\phi_2\rangle \to |\phi_3\rangle = \frac{i}{2\sqrt{2}}\{|000\rangle_1|010\rangle_2 + |001\rangle_1|011\rangle_2 + |010\rangle_1|011\rangle_2 + |011\rangle_1|010\rangle_2 + |100\rangle_1|100\rangle_2 + |101\rangle_1|101\rangle_2 + |110\rangle_1|101\rangle_2 + |111\rangle_1|100\rangle_2\}$.
- Apply the inverse gate, $\sqrt{-\mathrm{I}_2}^{\dagger}$, to each occupied atom of first register, we have $|\phi_3\rangle \to |\phi_4\rangle = \frac{1}{8}\sum_{w=000}^{111}|w\rangle_1\{(-1)^{000\cdot w}[1+(-1)^{s\cdot w}]|010\rangle_2 + (-1)^{001\cdot w}[1+(-1)^{s\cdot w}]|011\rangle_2 + (-1)^{100\cdot w}[1+(-1)^{s\cdot w}]|100\rangle_2 + (-1)^{101\cdot w}[1+(-1)^{s\cdot w}]|101\rangle_2\} = \frac{1}{4}\{[|000\rangle_1 + |011\rangle_1 + |100\rangle_1 + |111\rangle_1]|010\rangle_2 + [|000\rangle_1 - |011\rangle_1 + |100\rangle_1 - |111\rangle_1]|011\rangle_2 + [|000\rangle_1 + |011\rangle_1 - |100\rangle_1 - |111\rangle_1]|100\rangle_2 + [|000\rangle_1 - |011\rangle_1 - |100\rangle_1 + |111\rangle_1]|101\rangle_2\}$.

This example demonstrates that each two different states through $\wp_1$-site are mapped to only one state through $\wp_2$-site at $\gamma_1 = \gamma_2 = 0$. By measuring the first register states for each state across the second register, we will have four possible answers of w that are: $w_1 = 000$, $w_2 = 011$, $w_3 = 100$ and $w_4 = 111$. Then

$$s\cdot 000 = s_0 0 \oplus s_1 0 \oplus s_2 0 = 0\,(\text{No information given}). \tag{S40}$$

$$s\cdot 011 = s_0 0 \oplus s_1 1 \oplus s_2 1 = 0 \to s_1 \oplus s_2 = 0\,(\mathrm{mod}\ 2). \tag{S41}$$

$$s\cdot 100 = s_0 1 \oplus s_1 0 \oplus s_2 0 = 0 \to s_0 = 0\,(\mathrm{mod}\ 2). \tag{S42}$$

$$s\cdot 111 = s_0 1 \oplus s_1 1 \oplus s_2 1 = 0\,(\mathrm{mod}\ 2). \tag{S43}$$

Hence, the Eqs.(S41,S42,S43) are sufficient to determine that $s = 011$. Using the same procedures with the remaining schemes to decide and compute all possible values of s across each ideal oracle of Figs.(S3,S4,S5,S6). We find that the schemes with oracles of Figs.(S3a$_1$:S3a$_3$), Figs.(S3a$_4$:S3a$_6$), Figs.(S3a$_7$:S3a$_9$) and Figs.(S3a$_{10}$:S3a$_{12}$) decide that $s = 11$, $s = 10$, $s = 01$ and $s = 00$, respectively. Also, we find that Figs.(S4b$_1$:S4b$_5$), Figs.(S4b$_6$:S4b$_{10}$), Figs.(S4b$_{11}$:S4b$_{15}$), Figs.(S4b$_{16}$:S4b$_{20}$), Figs.(S5b$_{21}$:S5b$_{25}$), Figs.(S5b$_{26}$:S5b$_{30}$), Figs.(S5b$_{31}$:S5b$_{35}$) and Figs.(S6b$_{36}$:S6b$_{47}$) decide that $s = 001$, $s = 010$, $s = 011$, $s = 100$, $s = 101$, $s = 110$, $s = 111$ and $s = 000$, respectively.

Moreover, in the decimal notation, the outputs of the scheme in the presence of dissipation (OUD) with the oracle of Fig.(S4b$_{11}$) will be expressed as in the following:

$$\begin{aligned}\mathrm{OUD} \;=\; & \frac{1}{8}\{\sum_{q_1=0}^{7}\sum_{w=0}^{7}(-1)^{q_1\cdot w}\mathfrak{D}_0^{q_1}|w\rangle_1|0\rangle_2 + \sum_{q_2=0}^{7}\sum_{w=0}^{7}(-1)^{q_2\cdot w}\mathfrak{D}_1^{q_2}|w\rangle_1|1\rangle_2 \\ & + \sum_{q_3=0}^{7}\sum_{w=0}^{7}(-1)^{q_3\cdot w}\mathfrak{D}_2^{q_3}|w\rangle_1|2\rangle_2 + \ldots + \sum_{q_8=0}^{7}\sum_{w=0}^{7}(-1)^{q_8\cdot w}\mathfrak{D}_7^{q_8}|w\rangle_1|7\rangle_2\}.\end{aligned}$$

On measuring the first register, there will be eight possible values for the above four answers of w that are sufficient to decide for the correct value of s, $s \in \{000, 001, 010, 011, 100, 101, 110, 111\}$.

REFERENCES

1. O. Mandel, M. Greiner, A. Widera, T. Rom, T. W. Hänsch and I. Bloch, *Nature* **425** (2003) 937-940.
2. M. P. A. Fisher, P. B. Weichman, G. Grinstein and D. S. Fisher, *Phys. Rev. B* **40**(1989) 546.
3. Y.-J. Lin, K. Jiménez-García and I. B. Spielman, *Nature* **471**(2011) 83-86.
4. C. Zhang, *Phys. Rev. A* **82(R)** (2010) 021607.
5. Y. Zhang, L. Mao and C. Zhang, *Phys. Rev. Lett.* **108** (2012) 035302.
6. X.-J. Liu, M. F. Borunda, X. Liu and J. Sinova, *Phys. Rev. Lett.* **102** (2009) 046402.

7. J. R. Schrieffer and P. A. Wolff, Relation between the Anderson and Kondo Hamiltonians, *Phys. Rev.* **149** (1966) 491.
8. L.-M. Duan, E. Demler and M. D. Lukin, Controlling spin exchange interactions of ultracold atoms in optical lattices, *Phys. Rev. Lett.* **91** (2003) 090402.
9. A. B. Kuklov and B. V. Svistunov, Counterflow superfluidity of two-species ultracold atoms in a commensurate optical lattice, *Phys. Rev. Lett.* **90** (2003) 100401.
10. Y. Zhang, G. Chen and C. Zhang, *Sci. Rep.* **3** (2013) 1937.
11. T. Moriya, *Phys. Rev. Lett.* **4**(1960) 228 .
12. K. Zakeri, *J. Phys.: Condens. Matter* **29** (2017) 013001.
13. H. F. Wang, X. Q. Shao, Y. F. Zhao, S. Zhang and K. H. Yeon, *J. Phys. B: At. Mol. Opt. Phys.* **43** (2010) 065503.
14. A.-S. F. Obada, H. A. Hessian, A.-B. A. Mohamed and A. H. Homid, *J. Opt. Soc. Am. B* **30** (2013) 1178-1185.
